# The Role of Adiponectin and *ADIPOQ* Variation in Metabolic Syndrome: A Narrative Review

**DOI:** 10.3390/genes16060699

**Published:** 2025-06-10

**Authors:** Wiktoria Błażejewska, Justyna Dąbrowska, Joanna Michałowska, Paweł Bogdański

**Affiliations:** 1 Doctoral School, Poznan University of Medical Sciences, 60-812 Poznan, Poland; 88040@student.ump.edu.pl; 2 Department of Treatment of Obesity, Metabolic Disorders and Clinical Dietetics, Poznan University of Medical Sciences, 61-701 Poznan, Polandpbogdanski@ump.edu.pl (P.B.); 3 Department of Biochemistry and Molecular Biology, Poznan University of Medical Sciences, 60-781 Poznan, Poland

**Keywords:** adiponectin, obesity, metabolic syndrome, *ADIPOQ* gene, single-nucleotide variants

## Abstract

Metabolic syndrome (MetS), a significant global health concern, is characterized as a cluster of metabolic abnormalities that elevate the risk of type 2 diabetes mellitus (T2DM) and cardiovascular disease (CVD). Adiponectin, an adipokine secreted by adipose tissue, plays a crucial role in regulating glucose and lipid homeostasis while exhibiting protective effects against vascular alterations. Single-nucleotide variants (SNVs) in the *ADIPOQ* gene have significantly affected circulating adiponectin levels and metabolic parameters. This narrative review examines current evidence on the relationship between adiponectin, *ADIPOQ* gene variants, and metabolic syndrome. The findings indicate that lower adiponectin levels are associated with an increased risk of metabolic syndrome components, including elevated triglycerides (TGs), low-density lipoprotein cholesterol (LDL-C), and fasting glucose levels. In conclusion, adiponectin emerges as a key regulator of metabolic homeostasis, with SNVs in the *ADIPOQ* gene correlating with the development of metabolic-related complications.

## 1. Introduction

Metabolic syndrome can be considered a significant global health concern [1]. Its pathogenesis results from a multifaceted interaction of physiological, biochemical, clinical, and metabolic abnormalities, associated with an increased risk of atherosclerotic cardiovascular disease (ASCVD), T2DM, and premature mortality [2]. Over the past fifty years, the prevalence of metabolic disorders—including obesity, T2DM, hypertension, hyperlipidemia, and non-alcoholic fatty liver disease (NAFLD)—has risen dramatically worldwide. Metabolic syndrome is increasingly recognized as an early clinical marker that facilitates detecting and preventing these conditions [3,4,5]. The global prevalence of MetS ranges from 12.5% to 31.4%, depending on the diagnostic criteria. Regions with the highest rates are observed in Eastern Mediterranean territories and the Americas, and a country’s socioeconomic status plays a key role in its distribution [6]. The prevalence of metabolic syndrome shows an apparent linear increase from early adulthood until age 50, after which the rate tends to plateau. This pattern is influenced by other demographic factors such as gender and ethnic background [5]. Definition and diagnostic criteria for metabolic syndrome have evolved, reflecting continuous revisions by various international organizations [7]. In 2009, the International Diabetes Federation (IDF); American Heart Association (AHA); National Heart, Lung, and Blood Institute (NHLBI); World Heart Federation (WHF); International Atherosclerosis Society (IAS); and International Association for the Study of Obesity (IASO) reached a consensus on the diagnostic criteria for MetS. According to these guidelines, the presence of any three out of five specific abnormalities qualifies for diagnosis.

These criteria include the following:

⮚ elevated waist circumference (WC) (≥102 cm for men, ≥88 cm for women);
⮚ elevated fasting glucose (≥100 mg/dL) or ongoing treatment for dysglycemia;
⮚ elevated triglycerides (≥150 mg/dL) or ongoing treatment for hypertriglyceridemia;
⮚ reduced high-density lipoprotein cholesterol (HDL-C) (<40 mg/dL for men, <50 mg/dL for women) or ongoing treatment for low HDL-C;
⮚ elevated blood pressure (≥130/≥85 mm Hg) or ongoing treatment for hypertension [8].

These metabolic disturbances may significantly contribute to the development of type 2 diabetes, cardiovascular disease, and other chronic conditions, placing a growing burden on healthcare systems worldwide. Recent global data reveal that in 2019, obesity was linked to approximately 5 million deaths, hyperlipidemia to 4.3 million, T2DM to 1.4 million, hypertension to 1.1 million, and NAFLD to nearly 169,000 [3].

Adipose tissue, beyond its energy storage function, is an active endocrine organ. It regulates energy homeostasis by secreting bioactive adipokines such as leptin, resistin, and adiponectin [9,10], which act through endocrine, paracrine, and autocrine signaling pathways [5]. Furthermore, they affect several physiological processes, such as metabolism regulation, cardiovascular and immune system function, or modulation of inflammatory responses. In turn, dysregulation in their synthesis and/or secretion may negatively impact the balance between pro- and anti-inflammatory adipocytokines and lead to pathological conditions such as chronic inflammation and metabolic disorders [11].

Adiponectin, a protein hormone secreted by adipose tissue, was discovered in the late 1990s by four independent research groups. Initially, its clinical relevance remained unclear. The interest in this adipokine increased after its low serum levels were found to be correlated with obesity and greater visceral fat [5,12,13,14]. These relationships underscore the protective function of adiponectin and its potential role in the development of obesity-related metabolic disorders.

Adiponectin is encoded by the ADIPOQ (OMIM *605441) gene located on chromosome 3q27, which is considered a susceptibility locus for MetS, T2DM, and CVD [15,16]. Reduced adiponectin levels are associated with an elevated risk of metabolic syndrome components, and genetic factors appear to play a significant role in regulating these concentrations [17,18,19,20]. Over the past two decades, numerous studies have used linkage analyses [21,22,23], large-scale genome-wide association studies (GWASs), and meta-analyses to identify genetic factors influencing adiponectin levels and susceptibility to metabolic syndrome [22,24,25,26]. The studies identified single-nucleotide variants that are either causative or significantly associated with this metabolic disorder.

The growing global incidence of metabolic syndrome and related disorders underscores the importance of further research into molecular regulators such as adiponectin and its genetic determinants. This narrative review summarizes current knowledge on the role of adiponectin and ADIPOQ gene variants in metabolic disorders, providing a foundation for future research and potential clinical applications.

## 2. Adiponectin: Discovery, Structure, and Receptors

In 1995, after the discovery of leptin, the initial report on adiponectin was published. Analyzing mouse cell lines, the complementary DNA (cDNA) for this protein was sequenced and found to share homology with the complement component 1q (C1q), leading to its first name Adipocyte complement-related protein of 30 kDa (Acrp30) and later Adipocyte-derived Protein Q (AdipoQ). Further research, using large-scale random sequencing, identified the human cDNA for the adiponectin gene, as it was the most frequently encountered transcript in adipose tissue. This discovery led to its designation as the adipose most abundant gene transcript (apM1). Human adiponectin was further characterized by its isolation from plasma as a 28 kDa gelatin-binding protein, later termed GBP28 [10,13,27].

In plasma, adiponectin is primarily present as a full-length protein, with lower concentrations of a globular fragment resulting from proteolytic cleavage by leukocyte elastase [10]. Structurally, the full-length protein comprises an N-terminal signal sequence, a collagen-like domain, and a C-terminal globular domain. This globular domain exhibits structural homology to C1q, type VIII and X collagens, and tumor necrosis factor-alpha (TNF-α) [13,28]. Because of this structural organization, adiponectin forms a variety of oligomeric structures in both mouse and human serum, such as trimers, hexamers, and high-molecular-weight (HMW) multimers [10]. Interestingly, the globular form of adiponectin exhibits increased binding affinity for muscle cells and skeletal muscle membranes, whereas its interaction with hepatocytes and liver cell membranes is reduced. This differential binding is mediated by two identified adiponectin receptors, AdipoR1 and AdipoR2, which display distinct binding preferences for the globular and full-length forms of the adipokine. Notably, the amino acid sequence homology between human and murine variants of these receptors is remarkably high, reaching 96.8% for AdipoR1 and 95.2% for AdipoR2. The genes encoding these receptors, *ADIPOR1* (OMIM *607945) and *ADIPOR2* (OMIM *607946), are located on chromosomes 1p36.13-q41 and 12p13.33, respectively [29,30]. Both receptors play a crucial role in the regulation of glucose and lipid metabolism, as well as in modulating inflammatory responses and oxidative stress mechanisms [31].

In recent years, growing evidence has highlighted the therapeutic potential of adiponectin receptor agonists and genome-editing tools, such as CRISPR–Cas9, in addressing key features of metabolic syndrome. These strategies aim to restore impaired adiponectin signaling, a hallmark of obesity-related metabolic dysfunction [32,33].

AdipoRon and ADP355, a small molecule and peptide agonist, respectively, have been shown in preclinical models to activate AdipoR1 and AdipoR2 receptors. These compounds robustly stimulate AMP-activated protein kinase (AMPK) and peroxisome proliferator-activated receptor-α (PPAR-α) signaling pathways in the liver. In mouse models of non-alcoholic fatty liver disease and its inflammatory progression to non-alcoholic steatohepatitis (NASH), treatment with either compound reduced hepatic steatosis, lowered pro-inflammatory cytokine levels (TNF-α, IL-6), and improved insulin sensitivity and glucose tolerance. Notably, liver enzymes such as alanine transaminase (ALT) and aspartate aminotransferase (AST) also returned to near-normal levels, suggesting a reversal of hepatic injury [34].

Comparable effects have been reported with Tyr-Pro, a naturally occurring dipeptide that functions as a selective AdipoR1 agonist. Though still in the preclinical phase, Tyr-Pro enhances glucose uptake in skeletal muscle by increasing AMPK activation. In db/db mouse models of type 2 diabetes, this led to markedly improved glucose handling [35].

Another promising candidate, ALY688, is a synthetic ten-amino-acid peptide that closely mimics native adiponectin. Unlike earlier compounds, ALY688 has advanced into early clinical testing: Its sustained-release formulation (ALY688-SR) is currently being evaluated in a first-in-human Phase 1 trial for obesity-related conditions [36]. Preclinical data show that ALY688 activates AMPK in hepatocytes derived from human induced pluripotent stem cells (iPSCs) without inducing toxicity. In mouse models of fatty liver disease, it significantly reduced liver fat accumulation and inflammation, leading to lower NAFLD Activity Scores (NASs) [37].

## 3. Adiponectin and Metabolic Syndrome Components: Clinical Associations and Genetic Variants

**Figure 1:**
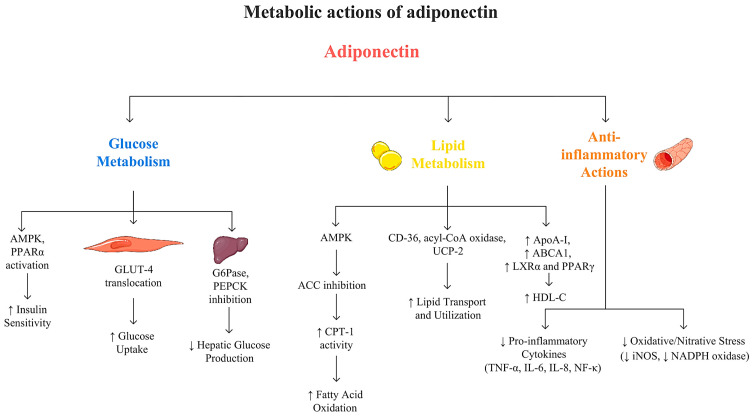
The diagram illustrates the pleiotropic effects of adiponectin on glucose and lipid metabolism and anti-inflammatory actions. In glucose metabolism, adiponectin enhances insulin sensitivity via adenosine monophosphate-activated protein kinase (AMPK) and proliferator-activated receptor-alpha (PPAR-α) activation, promotes glucose transporter-4 (GLUT-4) translocation to increase glucose uptake, and inhibits gluconeogenic enzymes—glucose-6-phosphatase (G6Pase) and phosphoenolpyruvate carboxykinase (PEPCK)—to reduce hepatic glucose production. In lipid metabolism, it stimulates fatty acid (FFA) oxidation through acetyl-CoA carboxylase (ACC) inhibition and carnitine palmitoyltransferase 1 (CPT-1) activation and enhances lipid transport/utilization by increasing cluster of differentiation 36 (CD-36), acyl-CoA oxidase, and uncoupling protein 2 (UCP-2). Adiponectin also elevates HDL-C levels via increased hepatic apolipoprotein AI (ApoA-I), ATP-binding cassette transporter A1 (ABCA1), liver X receptor alpha (LXRα), and peroxisome proliferator-activated receptor gamma (PPARγ) expression. Finally, its anti-inflammatory effects are mediated by suppression of pro-inflammatory cytokines: tumor necrosis factor alpha (TNF-α), interleukin-6 (IL-6), interleukin-8 (IL-8), nuclear factor kappa-light-chain-enhancer of activated B cells (NF-κB), and reduction in oxidative/nitrative stress through of nitric oxide synthase (iNOS) and nicotinamide adenine dinucleotide phosphate (NADPH) oxidase.

Adiponectin, an adipose-derived protein, plays a crucial role in regulating metabolic processes throughout the body by modulating lipid and carbohydrate homeostasis and protecting against vascular alterations [38,39] (Figure 1). As a key adipokine, adiponectin possesses insulin-sensitizing, anti-inflammatory, and anti-atherogenic properties, making it a vital player in metabolic health [40]. These effects are mediated, in part, by AdipoR1 and AdipoR2, which exhibit distinct tissue distributions and functions. Specifically, AdipoR1, predominantly expressed in skeletal muscles, enhances glucose uptake and oxidation, while AdipoR2, mainly found in the liver, regulates lipid metabolism and reduces hepatic gluconeogenesis. Through these mechanisms, adiponectin contributes significantly to metabolic balance [41,42]. Conversely, low adiponectin levels are strongly associated with increased risk of insulin resistance (IR) and elevated levels of triglycerides, total cholesterol (TC), and low-density lipoprotein, contributing to metabolic disturbances. Higher adiponectin concentrations correlate with improved glucose and lipid profiles, lower blood pressure, and reduced inflammatory markers. Consequently, adiponectin deficiency has been linked to an increased risk of metabolic syndrome components, including obesity, type 2 diabetes mellitus, hypertension, and cardiovascular diseases [40,43,44]. Given these correlations, adiponectin and its receptors have emerged as potential therapeutic targets. One example is AdipoRon, an adiponectin receptor agonist, which has been reported to improve metabolism in muscle, liver, and adipose tissue [45].

### 3.1. Central Obesity

Central obesity is defined as an excessive fat deposit in the abdominal region [46]. Visceral fat is a highly metabolically active organ, causing many metabolic alterations, including hypertriglyceridemia, increased availability of free fatty acids (FFAs), higher release of proinflammatory cytokines, and reduction in HDL cholesterol. Its excessive accumulation has been associated with insulin resistance, systemic inflammation, hyperlipidemia, and increased cardiovascular risk [46,47]. Therefore, abdominal obesity is a key risk factor of MetS and has been proposed as one of the diagnostic criteria for this condition. Waist circumference is used as a measure of visceral adiposity, and different thresholds are currently recommended based on population and ethnic group [8]. Adiponectin was first described as a protein produced exclusively in adipocytes [48]. However, further research showed that this adipokine is also produced by the skeletal muscles and can be expressed by the endothelial cells [49,50]. Initial research showed that the plasma concentration of adiponectin is negatively correlated with visceral adiposity [48,51]. This observation has been confirmed by the subsequent studies, and increased secretion of TNF-α, intermittent hypoxia, endoplasmic reticulum stress, mitochondrial dysfunction, and impaired leptin signaling occurring in obesity are among potential mechanisms responsible for a decrease in adiponectin level [51,52,53,54]. The distribution, expansion, and growth of adipose tissue (hypertrophy and/or hyperplasia) also play a role in adiponectin secretion and synthesis regulation [52]. A study by Drolet et al. showed that omental adipocyte adiponectin release is negatively correlated not only with total body fat mass but also with visceral adipose tissue (VAT) area and omental adipocyte diameter. Adiponectin release from subcutaneous cells was not associated with any measure of adiposity [55]. On the contrary, Meyer et al. indicated that subcutaneous adipose tissue (SAT) has a primary role in the determination of circulating levels of both total and HMW adiponectin [56]. A similar observation was made by Guenther et al., who confirmed that higher abdominal SAT, after adjustment for VAT, was associated with plasma adiponectin level, indicating that a higher SAT-to-VAT ratio may have a protective influence on T2DM and CVD risk [57]. An increase in the adiponectin expression can be achieved through various approaches. They include interventions associated with weight loss, such as caloric restriction, but also qualitative changes in the diet, including moderate alcohol consumption, the Mediterranean nutrition model, or appropriate omega-3 fatty acid intake [58]. Weight loss induced by non-dietary methods, including bariatric surgery and pharmacological treatment, was also associated with elevation of adiponectin levels. Yang et al. showed that a 21% reduction in body mass index (BMI) resulted in a 46% increase in plasma adiponectin concentration in obese subjects with gastric bypass surgery [59]. The increase in adiponectin levels after bariatric surgery can be observed independently of weight loss and is associated with improved function of fat tissue, a decrease in inflammation, and a reduction in insulin resistance [60]. Similar mechanisms can be observed in the pharmacological treatment of obesity. Meta-analysis of the impact of glucagon-like peptide receptor agonist (GLP-1RA) on adiponectin concentrations showed that this class of medication significantly increases the adiponectin level [61]. Wang et al. observed that GLP-1RA upregulates adiponectin expression both, in vitro and in vivo. In this study, GLP-1RA exendin-4 administration was associated with upregulation of adiponectin expression on mRNA and protein levels through SIRT1 and transcriptional factor FOXO-1 signaling pathways [62]. Another study also associated exendin-4 with increased adiponectin expression, and this observation was explained by the protein kinase A pathway in adipocytes [63]. Recently, the “adiponectin paradox” emerged in the literature as it was discovered that some patients with obesity exhibit high adiponectin concentrations without the beneficial effects of this adipokine [53,64]. This paradox can be explained by the alterations in the adiponectin clearance (that can be caused by renal or liver impairment), adiponectin resistance, and an increase in glycosylphosphatidylinositol phospholipase D (GPI-PLD) activity [53]. Kizer et al. suggested that in healthy individuals, low adiponectin levels are indeed associated with adverse events; however, in chronically ill patients, who experience cachexia and natriuretic peptide elevations, this association might be reversed [65]. Further well-designed studies are needed to understand the mechanisms behind this phenomenon fully. Association between obesity and adipokines is well established and includes anti-inflammatory adiponectin and proinflammatory leptin [66]. The ratio between those two proteins—adiponectin/leptin (A/L) ratio—has been proposed as a marker of adipose tissue dysfunction, where values ≥ 1 are considered normal, a ratio between 0.5 and 1.0 suggests moderate to medium increased risk, and values below 0.5 are indicative of a severe increase in cardiometabolic risk [67]. Low A/L ratio has been correlated not only with higher BMI, waist circumference, and adiposity but also with C-reactive protein (CRP) and serum amyloid A (SAA) levels. Therefore, this indicator could potentially explain the aetiopathogenesis of the MetS through the rise in the proinflammatory factors [68]. Interestingly, the recent study by Tarabeih et al. confirmed the association of the L/A ratio with MetS. However, their Additive Bayesian Network Analysis found the direct influence of monocyte to high-density lipoprotein cholesterol ratio (MHR), extracellular to intracellular water ratio (ECW/ICW), fat mass to body weight ratio, and age on MetS risk, but MetS seemed to be the cause of increased L/A ratio, not vice versa [69]. Considering many beneficial properties of adiponectin, its receptors—AdipoR1 and AdipoR2—have been proposed as potential targets for obesity treatment. AdipoRon, which is an adiponectin agonist, is a small compound that binds to AdipoR1 and AdipoR2 receptors. When administered in mice, it presented an action like the effects exerted by adiponectin, i.e., AMPK and PPAR-α pathways in muscles and liver. This resulted in improvement in insulin resistance and glucose intolerance in mice fed a high-fat diet (HFD). Moreover, administration of AdipoRon in genetically obese db/db mice ameliorated diabetes and prolonged the shortened lifespan. Therefore, authors summarized that synthetic AdipoR agonist could potentially reduce the consequences of excessive caloric intake and improve the treatment of obesity and related disorders, such as diabetes [70]. In the study by Iwabu et al., oral administration of AdipoRon in obese diabetic mice resulted in increased insulin sensitivity and exercise endurance; however, it did not significantly affect body weight or food intake [71]. Similar observations were made by Selvias et al., where mice fed HFD and receiving AdipoRon for almost a year showed better exercise endurance, insulin sensitivity, and smaller fatty infiltration in muscles and liver, in comparison to obese mice without AdipoRon administration. Even though AdipoRon did not affect body weight, the authors concluded that oral administration of this molecule promotes healthy aging and prevents the adverse metabolic effects of excessive caloric intake [72]. Other studies showed that oral administration of AdipoRon in mice can be effective in the treatment of different obesity comorbidities, including heart failure [73], depression [74], and inflammation [75,76]. Currently, other “adiponectin-based” therapies are under investigation, including monoclonal antibody activating adiponectin receptor [77] and novel AdipoRon agonist—AdipoAI [78]. Results may seem promising, but further studies, including double-blind randomized controlled trials (RCTs), are needed to determine if exogenous adiponectin administration could be a safe and effective treatment for obesity and related metabolic disorders in humans.

#### Adiponectin Gene Variants and Obesity Risk

Genetic variation in the ADIPOQ gene has been extensively studied due to its influence on circulating adiponectin levels and associated obesity risk. Swamy et al. comprehensively evaluated all 58 nonsynonymous single-nucleotide variants in the ADIPOQ gene (55 missense and three nonsense), employing multiple predictive algorithms (SIFT, PolyPhen-2, PROVEAN, I-Mutant 2.0, and MUpro) to identify variants likely to affect protein function and stability [79]. Their analysis identified 16 missense variants predicted to be both functionally damaging and structurally destabilizing. Five of these variants, including rs200573126, rs372597136, rs182223755, rs13061862, and rs138227502, are located within the N-terminal collagenous stalk, a helical region critical for initiating trimer formation and essential for proper adiponectin multimerization. The remaining 11 variants—rs199670988, rs199646033, rs62625753, rs121917815, rs79645624 (representing two distinct nucleotide substitutions: c.335G >C and c.335G > T), rs202043211, rs199547839, rs375480082, rs144526209, and rs199733477—cluster within the C-terminal globular C1q-like domain, which is responsible for stabilizing higher-order complexes and mediating receptor interactions. Since both domains are essential for the assembly and biological activity of high-molecular-weight adiponectin, amino acid substitutions within either domain may significantly impair hormone function and consequently influence obesity risk. Additionally, three nonsense variants—c.274C > T (p.Arg92Ter, rs139024247), c.635G > A (p.Trp212Ter, rs202013088), and c.658G>T (p.Glu220Ter, rs183590709)—are predicted to truncate the polypeptide upstream of crucial multimerization motifs, thus abolishing secretion of functional adiponectin [79]. Among these missense variants, the substitution p.Gly199Ser (c.595G > A, rs144526209) demonstrated the most significant predicted structural instability, suggesting the most severe potential functional impairment. However, this variant has not yet been characterized in clinical or experimental studies [79]. Functional studies previously established that specific amino acid substitutions in ADIPOQ, such as p.Gly84Arg (c.250G > A, rs199646033) and p.Gly90Ser (c.268G > A, rs62625753), impair high-molecular-weight multimer formation [19]. Additionally, variants such as p.Arg112Cys (c.334C > T, rs121917815) and p.Ile164Thr (c.491T > C, rs185847354) disrupt trimer assembly, leading to hypoadiponectinemia [10]. Furthermore, rare amino acid changes, including p.Pro32Leu (c.96C > G, rs201248773) and p.Arg55Cys (c.163C > T, rs138227502), decrease circulating adiponectin levels through dominant-negative effects [80]. Research data suggest that a significant proportion (30–70%) of the variability in circulating plasma adiponectin is affected by genetic factors [20]. While there is strong evidence that some SNVs are responsible for variation in plasma adiponectin concentration, the precise mechanisms underlying the association of *ADIPOQ* variation and metabolic disorders remain unclear. Some of the mechanisms proposed include inhibition of multimerization, production and secretion of adiponectin, and production of a truncated form of this protein [15]. So far, various SNVs have been associated with obesity risk in humans, whereas for some variants, available results are conflicting, or associations were not confirmed. Table 1 presents current research data investigating the role of *ADPOQ* variation in obesity.

### 3.2. Glucose and Lipid Profile

#### 3.2.1. Adiponectin and Glucose Metabolism

Type 2 diabetes mellitus is a multifactorial endocrine disorder characterized by chronic hyperglycemia, arising from insufficient insulin secretion, impaired insulin action, or a combination thereof [15,122]. This sustained elevation of blood glucose levels contributes to progressive dysfunction and eventual failure of multiple organ systems, notably the kidneys, cardiovascular system, and nervous tissue [15]. According to the International Diabetes Federation Diabetes Atlas, the global prevalence of diabetes among adults in 2019 was estimated at 9.3% (463 million individuals), with projections indicating an increase to 10.9% (700 million) by 2045; disproportionately higher rates are observed in urban and high-income regions [123]. These data underscore the intricate interplay between environmental and behavioral risk factors in the pathogenesis of T2DM.

Adiponectin, a key regulator of glucose metabolism, exerts anti-inflammatory and insulin-sensitizing effects [124]. Investigations indicate that elevated circulating adiponectin levels enhance insulin sensitivity [124,125], thereby potentially reducing T2DM risk [124]. Wang et al. demonstrated an inverse association between serum adiponectin concentrations and diabetes risk in a Chinese population [124]. Similarly, Yamamoto et al. supported the beneficial role of adiponectin in glucose tolerance, independently of visceral fat [125]. Furthermore, Lindberg et al. corroborated that increased adiponectin levels are positively correlated with decreased T2DM incidence and cardiovascular abnormalities [126]. These findings are consistent with other research, emphasizing adiponectin’s role in improving insulin sensitivity through various mechanisms in skeletal muscle and liver.

Adiponectin regulates glucose homeostasis through diverse metabolic pathways. For instance, it inhibits enzymes such as glucose-6-phosphatase (G6Pase) and phosphoenolpyruvate carboxykinase (PEPCK), leading to reduced hepatic gluconeogenesis and glycogenolysis [127]. Combs et al. confirmed this mechanism in a study on mice [128]. Moreover, hepatic glucose production may be limited by enhanced fatty acid oxidation [127]. Adiponectin promotes glucose utilization and fatty acid oxidation through activation of AMPK and PPAR-α pathways [129]. AMPK activation facilitates the translocation of glucose transporter-4 (GLUT-4) to the plasma membrane, thereby promoting glucose uptake in myocytes. Ceddia et al. demonstrated that treatment with globular adiponectin enhances GLUT-4 translocation and glucose uptake in skeletal muscle cells, reinforcing the role of adiponectin in maintaining glucose homeostasis [130]. Additionally, this protein has been reported to enhance glucose metabolism by stimulating lactate production, glucose oxidation, and glycogen synthesis [131]. Adiponectin protects pancreatic β-cells from ceramide- and cytokine-induced apoptosis. It supports insulin secretion through AdipoR1 and AdipoR2 activation, which promote ceramide de-acylation and formation of anti-apoptotic sphingosine 1 phosphate (S1P). This pathway enhances β-cell function [132,133]. Pro-inflammatory cytokines, such as TNF-α and interleukin-6 (IL-6), contribute to IR through impaired insulin signaling and glucose metabolism. Adiponectin may mitigate these effects by suppressing their expression and reducing activation of inflammatory mediators, including interleukin-8 (IL-8) and nuclear factor kappa-light-chain-enhancer of activated B cells (NF-κB), in endothelial cells. Additionally, it reduces oxidative and nitrative stress and helps maintain cellular insulin responsiveness by inhibiting inducible nitric oxide synthase and downregulating nicotinamide adenine dinucleotide phosphate (NADPH) oxidase expression [127].

In summary, adiponectin serves as an essential regulator of glucose metabolism and insulin sensitivity. Its ability to modulate multiple metabolic and inflammatory pathways highlights its therapeutic potential in the prevention of metabolic disorders such as type 2 diabetes or insulin resistance.

#### 3.2.2. Adiponectin and Lipid Metabolism

Adiponectin is a key regulator of lipid metabolism, enhancing fatty acid oxidation and maintaining lipid homeostasis across various tissues, including skeletal and cardiac muscles, the liver, and adipose tissue. These metabolic effects are primarily mediated through the activation of the AMPK and PPAR-α signaling pathways, subsequent to the binding of adiponectin to its receptors, AdipoR1 and AdipoR2 [28,127]. In skeletal muscle, adiponectin promotes lipid catabolism by activating AMPK, which phosphorylates and inhibits acetyl-CoA carboxylase (ACC). This inhibition reduces malonyl-CoA levels, thereby alleviating the inhibition of carnitine palmitoyltransferase 1 (CPT-1), the enzyme responsible for the mitochondrial import of fatty acids. Consequently, fatty acid oxidation is enhanced, supporting increased energy expenditure and improved lipid utilization [131]. Furthermore, adiponectin upregulates proteins that facilitate fatty acid transport and oxidation, such as fatty acid transporter/scavenger receptor, acyl-coenzyme A (acyl-CoA) oxidase, and uncoupling protein-2, further augmenting lipid catabolism within muscle cells [39].

Beyond its effects on fatty acid metabolism, adiponectin plays a crucial role in regulating the lipid profile. Yamamoto et al. demonstrated that serum adiponectin concentrations are positively correlated with HDL-C and negatively with TG levels, independent of BMI, in the Japanese population [134]. Similarly, Kangas-Kontio et al. confirmed a strong positive association between adiponectin levels and HDL-C concentrations in Finnish families [135]. One mechanism underlying the increase in HDL-C involves the upregulation of hepatic apolipoprotein AI (ApoA-I) synthesis and the stimulation of ATP-binding cassette transporter A1 (ABCA1) expression. Both processes are critical for cholesterol efflux and HDL particle formation and are likely mediated by the activation of nuclear receptors, such as liver X receptor alpha (LXRα) and peroxisome proliferator-activated receptor gamma (PPARγ) [136]. Another mechanism potentially explaining the positive correlation between adiponectin and increased HDL-C levels was demonstrated by Kobayashi et al., who reported an inverse association between serum adiponectin concentrations and hepatic lipase (HL) activity, independent of BMI and IR [137]. Regarding triglyceride metabolism, adiponectin facilitates the clearance of TG-rich lipoproteins, such as very low-density lipoproteins (VLDLs) and chylomicrons, by stimulating lipoprotein lipase (LPL), the enzyme responsible for hydrolyzing triglycerides in circulating lipoproteins. Adiponectin enhances both gene expression and enzymatic activity of LPL, particularly in skeletal muscle during fasting and in adipose tissue after food intake. Moreover, adiponectin appears to suppress apolipoprotein C-III (apoC-III), an inhibitor of LPL, and may enhance the expression of VLDL receptor (VLDLr) in skeletal muscle, thereby facilitating greater uptake and catabolism of VLDL particles [136].

These findings suggest that adiponectin not only enhances HDL synthesis but also improves the overall lipoprotein profile by promoting favorable changes in particle size, which are critical for cardiovascular protection.

#### 3.2.3. Adiponectin and Cardiovascular Health

Cardiovascular disease is a significant health concern, particularly affecting individuals over 50, and is marked by high rates of prevalence, disability, and mortality [138]. These conditions are frequently linked to modifiable risk factors such as obesity, hypertension, elevated LDL cholesterol, and diabetes [139]. Lifestyle choices, including diet, alcohol consumption, tobacco use, and physical activity, also play a crucial role in influencing cardiovascular risk [140].

Among the biological mechanisms linking these risk factors to cardiovascular outcomes, adiponectin has emerged as a key adipokine with protective properties. It exerts several beneficial effects related to glucose and lipid metabolism, including enhancing insulin sensitivity, diminishing oxidative stress and inflammation, improving endothelial cell function, and protecting myocardial cells [141]. Clinical studies have frequently observed an inverse correlation between adiponectin levels and cardiovascular risk, with hypoadiponectinemia identified as an independent risk factor for coronary artery disease [138]. However, some studies suggested that increased adiponectin levels may be associated with adverse cardiovascular outcomes, such as those with advanced heart failure [142]. Therefore, the role of adiponectin in cardiovascular disease is complex and may vary depending on the specific clinical context. Adiponectin plays a crucial role in maintaining vascular health by stimulating nitric oxide production via the PI3K and AMPK pathways. This process is essential for preserving endothelium-dependent vasodilation and counteracting atherosclerosis related to nitric oxide (NO) deficiency [143]. These vascular benefits are further supported by adiponectin-induced phosphorylation of endothelial nitric oxide synthase (eNOS) through both AMPK-dependent and AMPK-independent mechanisms [127,142]. Adiponectin also promotes angiogenesis through the interaction between AMPK and Akt signaling pathways; disrupting either pathway impairs eNOS activation and reduces endothelial cell migration, emphasizing the importance of this crosstalk [143,144]. Furthermore, adiponectin encourages endothelial cell survival, migration, and differentiation and reduces early atherogenesis by inhibiting monocyte adhesion to the endothelium via downregulation of resistin-induced E-selectin and vascular cell adhesion molecule-1 (VCAM) expression [143]. Adiponectin also protects against cardiovascular pathology by improving lipid metabolism, limiting foam cell formation, and inhibiting the proliferation of vascular smooth muscle cells, which are key processes in the development of atherosclerosis [138]. Adiponectin’s vascular effects are partly mediated by the activation of AdipoR1 and AdipoR2 receptors. Dysfunction of these receptors impairs adiponectin binding and action, potentially leading to elevated triglycerides, inflammation, and oxidative stress [142]. Adiponectin’s anti-inflammatory effects also involve the inhibition of inducible nitric oxide synthase and NADPH oxidase subunits, improving endothelial function and reducing oxidative and nitrative stress in vascular and renal tissues [143]. In animal models, such as apolipoprotein E-deficient (ApoE−/−) mice, administration of adiponectin increased the expression of anti-inflammatory factors (eNOS and IL-10) while reducing pro-inflammatory mediators (TNF-α, IL-6, and VCAM-1). This was accompanied by inhibition of the NF-κB signaling pathway and suppression of nuclear p65 expression, suggesting adiponectin’s critical role in modulating vascular inflammation and slowing atherosclerosis progression [145]. Furthermore, adiponectin promotes a phenotypic switch in macrophages from a pro-inflammatory M1 state to an anti-inflammatory M2 state, which reduces plaque formation and supports the stabilization of existing atherosclerotic lesions [143].

In heart failure, adiponectin exhibits a cardioprotective role by mitigating oxidative stress, ischemia–reperfusion injury, and pathological myocardial remodeling. It possesses anti-apoptotic, anti-inflammatory, and antihypertrophic properties, inhibits interstitial fibrosis, and promotes angiogenesis [142]. Through AMPK activation, adiponectin protects cardiomyocytes under hypoxia-reoxygenation conditions and may prevent hypertrophy via inhibition of endothelin-1- and α-adrenergic-induced ERK signaling [142,143]. Interestingly, while adiponectin generally has protective effects, large-scale observational research has shown that elevated plasma adiponectin is associated with an increased risk of heart failure and other cardiovascular events. However, Mendelian randomization analyses do not support a causal relationship, suggesting these associations may be confounded by other factors or reflect a compensatory response to cardiovascular stress [146]. These findings highlight the complexity of adiponectin’s role in advanced heart disease and the need for nuanced interpretation of elevated levels in clinical contexts.

Adiponectin also reduces hepatic glucose production and enhances peripheral glucose and fatty acid utilization, thereby improving insulin sensitivity and countering hyperglycemia, both critical risk factors for vascular disease [127]. In conditions such as obesity and insulin resistance, adiponectin levels decline, diminishing its protective effects and increasing disease susceptibility [142]. It also modulates lipid profiles by raising HDL-C and lowering triglycerides through mechanisms involving increased LPL activity, enhanced VLDL receptor expression, and suppression of apo-CIII [127]. These combined metabolic and vascular actions reinforce adiponectin’s protective influence in cardiometabolic disorders.

#### 3.2.4. The Role of Adiponectin Gene Variants in Modulating Lipid and Carbohydrate Profiles

Large-scale genome-wide association studies have expanded our understanding of the polygenic basis of T2DM. In a comprehensive meta-analysis, Mahajan et al. identified 243 genome-wide significant loci (encompassing 403 distinct association signals following conditional analyses), reflecting the extensive genetic heterogeneity observed in T2DM [147]. Type 2 diabetes mellitus is often accompanied by dyslipidemia, characterized by an imbalance in lipid profiles. This typically involves elevated levels of LDL-C, TC, and TG, coupled with reduced HDL-C. These lipid abnormalities not only exacerbate insulin resistance but also significantly increase the risk of cardiovascular complications, such as coronary heart disease and atherosclerosis [148,149]. Accumulating evidence suggests that SNVs within the *ADIPOQ* gene are associated with modulating adiponectin levels and, by extension, metabolic homeostasis. Among the most extensively studied variants in *ADIPOQ*, rs2241766 (T > G), rs266729 (C > G), and rs1501299 (G > T) have been repeatedly linked to altered adiponectin levels, adverse lipid profiles, and increased susceptibility to T2DM or its complications [150,151,152]. Specifically, rs2241766 (a synonymous coding variant) and rs266729 (a promoter variant) often correlate with reduced adiponectin concentrations and an elevated risk of insulin resistance or cardiovascular comorbidities. By contrast, rs1501299 (an intronic variant) demonstrates population-specific effects, conferring atherogenic lipid abnormalities in some contexts while exhibiting a possible protective influence on adiponectin levels in others [151]. In addition to these well-characterized variants, several 3′UTR SNVs, including rs1063537, rs1063538, and rs2082940, have gathered increasing attention for their potential to modulate *ADIPOQ* mRNA stability and thus protein production [85,150,153,154]. Several studies have linked rs1063537 and rs1063538 to an increased risk of T2DM in certain populations [150,153], while rs2082940 has been associated with a higher probability of progression from impaired glucose tolerance (IGT) to T2DM in Finnish cohorts [79]. Although the impact of these 3′UTR variants varies across populations, collectively they underline the importance of post-transcriptional regulation in adiponectin-mediated metabolic processes. Furthermore, ADIPOR1 and ADIPOR2, which encode the primary adiponectin receptors, also affect glycemic control and lipid metabolism [42]. Genetic variants in these receptors also influence metabolic outcomes. For instance, Shramko et al. demonstrated that rs2275737 in ADIPOR1 correlates with elevated HbA1c levels, whereas rs16928751 in ADIPOR2 increased T2DM risk in a Russian cohort [155].

Given adiponectin’s multifaceted role in glucose and lipid metabolism, as well as its genetic regulation, understanding the associations between *ADIPOQ* polymorphisms and metabolic traits is crucial. The following Table 2 presents a summary of key studies investigating the effects of *ADIPOQ* gene variants on glucose homeostasis and lipid profiles.

**Table 2:** Impact of *ADIPOQ* gene variants on lipid and glucose profile.

rsID	Allele Change	Variant Location/Molecular Consequences	Observed Associations	Study Population	References
rs1501299	G > T	Intron	- G allele significantly associated with increased T2DM risk, strongest in the additive model (OR = 1.09, 95% CI 1.03–1.15, *p* = 0.005) and recessive model (OR = 1.09, *p* = 0.03)	- East-Asian adults from a meta-analysis of 16 studies: T2DM cases (n = 6115) and controls (n = 7155)	[156]
			- No association was found between SNV and T2DM in the overall meta-analysis	- Asian, Caucasian, Pima, Iranian, South-African, and Mexican adults from a meta-analysis of 22 studies: T2DM cases (n = 7958) and controls (n = 18,765)	[157]
			- GG genotype was associated with increased risk of IR and T2DM; lower adiponectin levels among individuals with G allele (GG = 10.4 ± 0.85 μg/mL, GT = 13.7 ± 0.87 μg/mL, TT = 16.6 ± 2.24 μg/mL, *p* = 0.01) in subjects with higher BMI	- Japanese adults: T2DM cases (n = 384) and controls (n = 480)	[158]
			- G allele associated with higher IGT risk (OR = 1.65, 95% CI 1.08–2.51, *p* = 0.020); no association with T2DM, no association with BMI, WC, WHR, fasting glucose, fasting insulin, HOMA-IR, HDL-C, TG, or serum adiponectin	- Spanish adults (n = 747)	[159]
			- G allele carriers were associated with elevated TG, LDL-C, and HOMA-IR; lower adiponectin levels were observed among individuals with the GG genotype when compared to the TT genotype (6.22 μg/mL vs. 7.67 μg/mL, respectively; p < 0.05)	- Korean non-diabetic adults (n = 902)	[160]
			- T allele significantly associated with higher T2DM risk (OR = 1.31, 95% CI 1.04–1.65, *p* = 0.002)	- Chinese adults: T2DM cases (n = 340) and controls (n = 340)	[161]
			- Significantly associated with T2DM under an additive model after adjustment for age, sex, and BMI; TT genotype associated with higher TC (*p* = 0.009), LDL-C (*p* = 0.002), and TG (*p* = 0.03), and with lower HDL-C (*p* = 0.003)	- South-Indian adults: T2DM cases (n = 1100) and controls (n = 1100)	[92]
			- Significantly associated with T2DM (*p* < 0.05)	- Saudi adults: T2DM cases (n = 96) and controls (n = 96)	[162]
rs2241766	T > G	Exon/synonymous variant	- No association with T2DM in the overall meta-analysis; neither TG nor GG genotypes showed a significant association, and no ethnic-specific association was detected despite differences in G-allele frequency across populations	- Asian and European adults from a meta-analysis of 28 studies: T2DM cases (n = 9701) and controls (n = 17,193)	[163]
			- No statistically significant association with T2DM	- Asian and European adults from a meta-analysis of 21 studies: T2DM cases (n = 6370) and controls (n = 15,443)	[157]
			- Significantly associated with increased risk of diabetic dyslipidemia; G allele comparison vs. healthy controls yielded the highest OR = 4.20 (95% CI 2.45–7.22, *p* < 0.0001)	- Asian Indian adults: diabetes with dyslipidemia (n = 88), diabetes without dyslipidemia (n = 86) and healthy controls (n = 90)	[164]
			- Significantly associated with increased T2DM risk in carriers of the G/T and G/G genotypes (G/G vs. T/T: OR = 1.70, 95% CI 1.09–2.65, *p* = 0.02; G/T vs. T/T: OR = 1.41, 95% CI 1.06–1.88); among individuals with T2DM, the variant was also associated with a higher risk of CAD	- Japanese adults: T2DM cases (n = 384) and controls (n = 480)	[158]
			- Associated with T2DM; G allele protective (G vs. T: OR = 0.82, 95% CI 0.70–0.95, *p* = 0.009; GG vs. TT: OR = 0.61, 95% CI 0.42–0.89, *p* = 0.025)	- Taiwanese adults: T2DM cases (n = 570) and controls (n = 1700)	[153]
			- G allele associated with higher IGT risk (OR = 1.65, 95% CI 1.08–2.51, *p* = 0.020); no association with T2DM; BMI, WC, WHR, fasting glucose, fasting insulin, HOMA-IR, HDL-C, TG, or serum adiponectin	- Spanish adults (n = 747)	[159]
			- Significantly associated with higher T2DM risk: GG genotype (~5-fold vs. TT, age/sex-adjusted) and TG genotype (~2-fold); GG and TG also associated with higher TC, TG, insulin, and HOMA-IR compared with TT	- Iraqi adults: T2DM cases (n = 420) and controls (n = 420)	[165]
			- G allele associated with higher T2DM risk (dominant model TG + GG vs. TT: HR = 1.63, 95% CI 1.04–2.58, *p* = 0.035) and TT genotype linked to lower serum adiponectin (marginally significant, *p* = 0.056)	- Finnish adults with IGT, all overweight (n = 507)	[85]
rs266729	C > G	Promoter	- G allele was associated with an increased risk of T2DM; the additive genetic model shows OR = 1.13 with a 95% CI= 1.06–1.19, *p*-value < 0.001	- Asian, European, African, and American adults from a meta-analysis of 7 studies: T2DM cases (n = 5948) and controls (n = 6395)	[166]
			- G allele was associated with an increased risk of T2DM (OR = 1.07 (95% CI 1.03–1.11, *p* = 0.001) particularly in Caucasian populations	- Asian and European adults from a meta-analysis of 6 studies: T2DM cases (n = 6127) and controls (n = 12,097)	[157]
			- G allele significantly associated with higher T2DM risk (allelic OR = 1.15, 95% CI 1.06–1.25, *p* = 0.001; recessive OR = 1.45, 95% CI 1.18–1.77, *p* = 0.0004); G is the risk allele	- Adults from multiple populations in a meta-analysis (n = 6425)	[167]
			- Significantly associated with T2DM under additive, dominant, and recessive models (*p* < 0.001)	- Tunisian Arab adults: T2DM cases (n = 917) and controls (n = 748)	[168]
			- Significantly associated with T2DM under an additive model after adjustment for age, sex, and BMI; GG genotype linked to higher fasting plasma glucose (*p* = 0.001), HbA1c (*p* = 0.007), and TG (*p* = 0.03)	- Indian adults: T2DM cases (n = 1100) and controls (n = 1100)	[92]
rs17366743	T > C	Exon/missense variant	- C allele was associated with an increased risk of T2DM (HR= 1.94, 95% CI 1.16–3.25, *p* = 0.01 in additive model); C allele was also associated with higher mean fasting plasma glucose over 28 years of follow-up (*p* = 0.0004)	- Framingham offspring study: predominantly European-ancestry men and women from the USA (n = 2543) followed for 28 years (1971–2002)	[114]
			- No association with T2DM and adiponectin level	- French Caucasians (n = 1373 unrelated adults) + family extension (n = 743 from 148 T2DM families)	[19]
rs7627128	C > A	Intron	- Minor A allele in men associated with lower HOMA-IR	- African-American adults with genetic data (n = 3020)	[117]
			- A allele significantly associated with higher T2DM risk (OR = 1.43, 95% CI 1.17–1.98, *p* = 0.002)	- Chinese adults: T2DM cases (n = 340) and controls (n = 340)	[161]
rs17300539	G > A	Promoter	- A allele showed borderline association with increased T2DM risk overall (OR = 1.08, 95% CI 1.00–1.16); association was significant only in Europeans (OR = 1.14, 95% CI 1.03–1.26)	- Asian, European, and mixed-ethnicity adults from a meta-analysis of 15 studies: T2DM cases (n = 10,158) and controls (n = 19,148)	[163]
			No significant association with T2DM	- European adults from a meta-analysis of 12 studies: T2DM cases (n = 4139) and controls (n = 12,530)	[157]
			- A allele associated with higher adiponectin (*p* < 0.0001) and with lower T2DM risk (OR = 0.79, 95% CI 0.60–1.06, *p* = 0.030); A is the protective allele	- French Caucasians (n = 1373 unrelated adults) + family extension (n = 743 from 148 T2DM families)	[19]
			- Associated with lower adiponectin and higher T2DM risk; GG genotype linked to higher HOMA-IR, insulin and TG levels, and increased IR and MetS risk	Spanish adults (n = 180)	[169]
			- Significantly associated with T2DM under additive, dominant, and recessive models (*p* < 0.001)	- Tunisian Arab adults: T2DM cases (n = 917) and controls (n = 748)	[168]
			- Significantly associated with T2DM under an additive model after adjustment for age, sex, and BMI	- Indian adults: T2DM cases (n = 1100) and controls (n = 1100)	[92]
rs182052	G > A	Intron	- No association with T2DM, fasting glucose, insulin, HOMA-IR, or blood lipid profiles	- European, African-American, and East-Asian adults from a meta-analysis of 33 studies (26 European-descent cohorts and 7 non-European cohorts): T2DM cases (~22,000) and controls (~58,000)	[170]
			- A allele significantly associated with higher T2DM risk (OR = 1.28, 95% CI 1.05–1.59, *p* = 0.022)	Chinese adults: T2DM cases (n = 340) and controls (n = 340)	[161]
			- Significantly associated with T2DM (OR = 1.40, 95%CI 1.17–1.67 for dominant model)	African-American adults: T2DM with ESRD (n = 851), T2DM without nephropathy (n = 317) and controls (n = 871)	[171]
rs17846866	T > G	3′UTR	- GG genotype associated with higher fasting glucose in controls (*p* < 0.001); no association with T2DM risk	Kazakh adults: T2DM cases (n = 136) and controls (n = 577)	[103]
			- Associated with increased risk of hypoadiponectinemia, the mean serum adiponectin levels lower in subjects with GG (4.99 ug/mL, *p* = 0.007 for controls; 4.54 μg/mL, *p* = 0.017 in diabetics) and TG (6.00 μg/mL, *p* = 0.001 for controls; 4.75 μg/mL, *p* < 0.0001 in diabetics) compared to TT (7.31 μg/mL in controls; 6.03 μg/mL for diabetics); frequency of G allele was significantly higher in T2DM patients (*p* < 0.001)	Asian-Indian adults: T2DM cases (n = 2000) and controls (n = 2000)	[94]
			Both heterozygous (TG vs. TT; OR = 1.92; 95% CI 1.01–3.66; *p* = 0.004) and homozygous variant (GG vs. TT; OR = 4.83; 95% CI 1.50–15.55; *p* = 0.004) was associated in T2DM	Saudi adults: T2DM cases (n = 96) and controls (n = 96)	[162]
rs3774261	A > G	Intron	- No association with T2DM risk in a case–control study; G allele was significantly associated with higher circulating adiponectin (meta-analysis, *p* ≤ 0.02)	European-Australian adults from a meta-analysis combining BHS, CUDAS, and FDS: T2DM cases (n = 967) and controls (n = 2355)	[172]
			- Significantly associated with T2DM under an additive model after adjusting for age, sex, and BMI	South-Indian adults: T2DM cases (n = 1100) and controls (n = 1100)	[92]
			- GG genotype associated with increased risk of T2DM in participants with a BMI <24, compared with the AA genotype (adjusted OR = 1.64, 95% CI 1.41–2.46; *p* = 0.03)	Chinese Han adults from northeast China: T2DM cases (n = 993) and controls (n = 966)—total n = 1959	[173]
rs822393	C > T	Intron	- Significantly associated with T2DM under an additive model after adjustment for age, sex, and BMI; associated with lower adiponectin levels	South-Indian adults: T2DM cases (n = 1100) and controls (n = 1100)	[92]
			- T allele associated with higher clamp-derived insulin sensitivity in whites (TT > CT > CC; *p* = 0.0034)	- U.S. cohort of white parents (n = 235) and offspring (n = 349) plus African-American parents (n = 27) and offspring (n = 92); total n = 703	[174]
			- T allele associated with low HDL-C (OR = 2.75, 95% CI 1.87–4.02, *p* = 0.03)	Caucasian obese adults from Spain (n = 1004)	[175]
			- T allele was associated with lower HDL-C under dominant (*p* = 0.0024), codominant (*p* = 0.00036), recessive (*p* = 0.00085), and additive models (*p* = 0.00018); T allele was also associated with lower ApoA1 under dominant (*p* = 4 × 10⁻^4^), codominant (*p* = 0.00013), recessive (*p* = 0.00134), and additive models (*p* = 4 × 10⁻^5^)	- European adolescents aged 12–18 years (n = 1057)	[176]
			- No association with T2DM; T allele linked to higher HbA1c in controls (TT > CT > CC; *p* = 0.043)	- Chinese Han adults from northeast China: T2DM cases (n = 993) and controls (n = 966)—total n = 1959	[173]
rs822395	C > A	Intron	A allele was associated with lower ApoA1 under dominant (*p* = 0.00234), codominant (*p* = 0.00123), recessive (*p* = 0.00314), and additive models (*p* = 0.00027); A allele was also associated with higher CVD risk under recessive (*p* = 0.00234) and additive models (*p* = 0.00238)	European adolescents aged 12–18 years (n = 1057)	[176]
rs822396	G > A	Intron	- Significant association with T2DM under dominant genetic models (*p* = 0.02)	North-African Tunisian Arab adults: T2DM cases (n = 917) and controls (n = 748)	[168]
			- Allele significantly associated with higher T2DM risk (GG vs. AG + AA, OR = 3.63, 95% CI 1.20–10.96, *p* = 0.022); GG genotype linked to higher TC, TG, LDL-C, and VLDL	North-Indian Punjabi adults: T2DM cases (n = 316) and controls (n = 300)	[177]
			- Significantly associated with T2DM under an additive model after adjustment for age, sex, and BMI; GG genotype associated with higher fasting plasma glucose (*p* = 0.05) and higher total cholesterol (*p* = 0.0001)	South-Indian adults: T2DM cases (n = 1100) and controls (n = 1100)	[92]
rs16861205	G > A	Intron	- No significant association with T2DM	African-American adults: T2DM cases (n = 586) and controls (n = 2434)	[117]
rs7649121	A > T	Intron	- T allele associated with lower T2DM risk (adjusted OR = 0.79, 95% CI 0.66–0.95 for AT; OR = 0.80, 95% CI 0.67–0.96 for AT/TT vs. AA)	Chinese Han adults: T2DM cases (n = 1105) and controls (n = 1107)	[178]
			- T allele was significantly associated with lower HDL-C under dominant (*p* = 0.00011), codominant (*p* = 0.00010), overdominant (*p* = 0.00340), and additive (*p* = 0.00002) models; T allele was also associated with lower ApoA1 under dominant (*p* = 0.00019), codominant (*p* = 0.00026), overdominant (*p* = 0.00347), and additive (*p* = 0.00005) models	European adolescents aged 12–18 years (n = 1 057)	[176]
rs1063537	C > T	3′UTR	- T allele was significantly associated with lower T2DM risk (OR = 0.64, *p* = 0.014)	Chinese Han adults: T2DM cases (n = 188) and controls (n = 176)	[150]
			- C allele associated with higher risk of diabetic nephropathy in Taiwanese men with T2DM (CC vs. CT + TT: HR = 1.89, 95% CI 1.15–3.11, *p* = 0.013; additive model HR = 1.73, *p* = 0.013); no association in women	Taiwanese adults with T2DM and normoalbuminuria at baseline, prospective cohort (n = 566; 263 men, 303 women; 6-year follow-up)	[154]
rs1063538	T > C	3′UTR	- TT genotype was associated with lower T2DM risk; CC genotype associated with higher T2DM risk	Taiwanese adults: T2DM cases (n = 570) and controls (n = 1700)	[153]
			- Significantly associated with T2DM under a dominant genetic model (*p* = 0.02)	North-African Tunisian Arab adults: T2DM cases (n = 917) and controls (n = 748)	[168]
rs16861194	A > G	Promoter	- G allele associated with higher T2DM risk, mainly in Europeans (additive model: OR = 1.15, 95% CI 1.04–1.27)	European and Asian adults from a meta-analysis of 39 studies: T2DM cases (n = 3176) and controls (n = 4007)	[163]
			- Significant association with T2DM under additive and dominant models (p < 0.001)	North-African Tunisian Arab adults: T2DM cases (n = 917) and controls (n = 748)	[168]
rs2082940	T > C	3′UTR	- TT genotype associated with higher risk of progression from IGT to T2DM vs. CC (HR = 5.37, 95% CI 1.31–22.00, *p* = 0.020); baseline adiponectin lower in CC carriers, especially in men (sex–genotype interaction *p* = 0.056)	- Finnish adults with IGT and overweight (n = 507)	[85]

**Abbreviations: *ADIPOQ***—adiponectin gene; **ApoA1**—apolipoprotein A1; **BMI**—body mass index; **CAD**—coronary artery disease; **CI**—confidence interval; **CRP**—C-reactive protein; **CVD**—cardiovascular disease; **DBP**—diastolic blood pressure; **FPG**—fasting plasma glucose; **HbA1c**—glycated hemoglobin; **HDL-C**—high-density lipoprotein cholesterol; **HOMA-IR**—Homeostatic Model Assessment of Insulin Resistance; **HR**—hazard ratio; **IGT**—impaired glucose tolerance; **IR**—insulin resistance; **LDL-C**—low-density lipoprotein cholesterol; **MAP**—mean arterial pressure; **MetS**—metabolic syndrome; **OR**—odds ratio; **SAT**—subcutaneous adipose tissue; **SBP**—systolic blood pressure; **SNV**—single-nucleotide polymorphism/variant; **T2DM**—type 2 diabetes mellitus; **TC**—total cholesterol; **TG**—triglycerides; **UTR (3′)**—3′ untranslated region; **VAT**—visceral adipose tissue; **VLDL**—very-low-density lipoprotein; **WC**—waist circumference; **WHR**—waist-to-hip ratio.

### 3.3. Hypertension

Hypertension, i.e., elevated blood pressure, remains the leading cause of death globally, accounting for over 10 million deaths each year [179]. This condition is diagnosed when systolic blood pressure (SBP) is equal to or higher than 140 mm Hg and/or diastolic blood pressure (DBP) is ≥90 mm Hg. Whenever possible, a diagnosis should not be made using a single measurement, unless BP measurement is ≥180/110 mmHg, and there is evidence of cardiovascular disease [180]. Hypertension usually coexists with other metabolic disorders, including obesity, T2D, and dyslipidemia [181]. It is estimated that in 2010, over 30% of the global population had this disease, and its prevalence was higher in low- and middle-income countries [182]. Various factors have been identified and associated with elevated blood pressure. Those include modifiable risk factors, including unhealthy diet, low physical activity, alcohol and tobacco use, and non-modifiable factors, including age, medical history, and genetic predisposition [183].

Various studies showed that hypertensive populations have lower adiponectin levels and that the concentration of this adipokine is inversely associated with the risk of elevated BP [184,185,186]. In a prospective study by Chow et al., adiponectin was found to be a significant independent predictor of incident hypertension. Normotensive subjects with the lowest baseline serum adiponectin levels had an increased risk of becoming hypertensive [186]. Similar results were presented in the study by Li et al., where subjects with hypertension had significantly lower plasma adiponectin levels than those without hypertension. However, in the subjects who were normotensive, adiponectin level correlated negatively with SBP only if obesity was present. Therefore, the authors concluded that the relationship between plasma adiponectin level and blood pressure depends on the presence of obesity [187]. Meta-analysis of Kim et al. revealed that every 1 µg/mL increase in adiponectin level was associated with a 6% reduced risk of hypertension [184]. Available research suggests that adiponectin may regulate blood pressure via brain-mediated and endothelium-mediated mechanisms. Animal studies showed that adiponectin plays a significant role in BP regulation by increasing endothelial nitric oxide (NO) production, improving endothelial function, suppressing sympathetic nervous system activity, and promoting anti-inflammatory macrophage phenotypes [184]. In humans, hypoadiponectinemia was associated with a lower vasodilatory response, whereas administration of adiponectin was associated with increased production of NO in the aortic endothelial cells. Various mechanisms have been proposed that explain the positive effect of adiponectin on NO synthase, including activation of the AMPK pathway, increased mRNA stability, Ser1179 phosphorylation, and enhancing the association with the heat-shock protein 90 (Hsp90). Moreover, adiponectin may promote endothelial cell function via cyclooxygenase-2 (COX-2)-dependent mechanisms [188,189]. Renin–angiotensin–aldosterone system (RAAS), which is a key factor in regulating BP, might also be associated with adiponectin levels. Studies showed that the interventions with an angiotensin II receptor blocker or angiotensin-converting enzyme inhibitor were associated with high adiponectin levels in hypertensive patients [190,191,192].

#### The Influence of Adiponectin Gene Variations on Hypertension

Various variants of the *ADPIOQ* gene have been associated with low levels of adiponectin [114], whereas inconsistent results are present for genetic variation of *ADIPOQ* and hypertension risk [193]. Two independent meta-analyses found that rs2241766 is associated with the risk of hypertension, whereas results for rs1501299 and rs266729 were conflicting [193,194]. In the study of Fan et al., the minor G allele of rs2241766 was associated with an increased risk of hypertension by 16%, whereas the GG genotype was associated with an increased risk of this disease by 34% [193]. Similarly, Yu et al. showed that the G allele of this variant was associated with an increased risk of hypertension by 10% [194]. On the other hand, in the meta-analysis investigating the Chinese population alone, this variant was marginally associated with hypertension but only under the recessive model (GG vs. GT/TT: OR = 1.22, 95% CI 1.01–1.48) [195].

Conflicting results for other variants may be associated with various factors, including race, environmental factors, and age differences. Further studies investigating large, homogenous groups are needed to determine which variants are associated with hypertension risk in different populations. Table 3 presents a summary of the current studies investigating the association between genetic variation of *ADIPOQ* and blood pressure.

**Figure 2:**
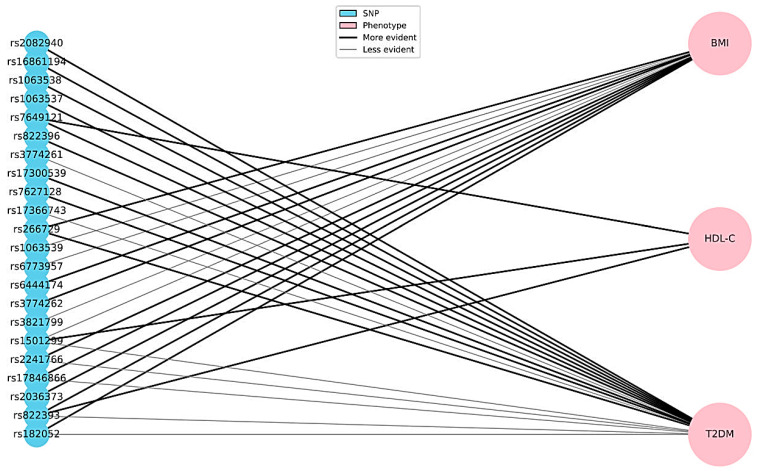
Bipartite network illustrating associations between *ADIPOQ* gene SNVs (blue nodes) and metabolic phenotypes (light pink nodes): BMI, HDL-C, and T2DM. Edge color and thickness reflect the strength of association based on literature evidence: Black edges represent more consistent findings, while gray edges indicate less consistent or preliminary associations. Node sizes distinguish SNVs from phenotypes.

**Figure 3:**
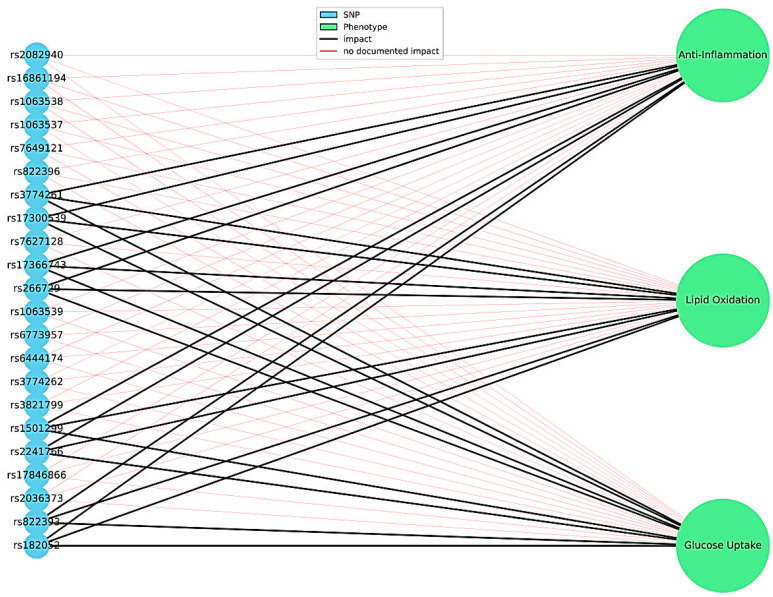
Bipartite network illustrating associations between *ADIPOQ* gene SNVs (blue nodes) and metabolic phenotypes (light green nodes): glucose uptake, lipid oxidation, and anti-inflammatory effects. Edge color and width indicate the presence or absence of documented effects based on literature: Black edges represent SNVs with reported impact; red edges indicate no documented association. Node size differentiates SNVs and phenotypes.

Figure 2 and Figure 3 provide a graphical summary of the associations between *ADIPOQ* gene SNVs and metabolic phenotypes (BMI, HDL-C, and T2DM), as well as the impact of individual variants on metabolic processes such as glucose uptake, lipid oxidation, and anti-inflammatory responses.

## 4. Epigenetic Regulation of ADIPOQ Gene

Many independent studies showed that multiple epigenetic mechanisms regulate *ADIPOQ* expression. The primary regulatory mechanism includes hypermethylation of the *ADIPOQ* promoter and regulatory regions [203,204,205], which are reinforced by repressive histone marks [206] and specific miRNA binding [207,208].

### 4.1. ADIPOQ Promoter Hypermethylation

Kim et al. demonstrated that obesity, chronic overnutrition, and inflammatory signals drive DNMT1-dependent hypermethylation of a distal promoter region (R2), located about 1 kb upstream of the *ADIPOQ* transcription start site. Kim et al. showed that hypermethylation of R2 silences *ADIPOQ* transcription and increases insulin resistance. Significantly, pharmacological inhibition or siRNA-mediated knockdown of DNMT1 reverses this hypermethylation, restoring adiponectin expression and improving glucose tolerance [204]. Kim et al. also noted that two SNVs, rs17300539 (−11391 G>A) and rs266729 (−11377 C>G), reside within the same distal regulatory region (R2). Interestingly, several earlier studies have reported that these promoter variants significantly correlate with circulating adiponectin levels [118,209,210]. Specifically, individuals with the AA genotype at rs17300539 have higher adiponectin levels than GG homozygotes. Likewise, individuals with the GG genotype at rs266729 have lower adiponectin levels than CC carriers. Since each variant alters a CpG dinucleotide, Kim et al. proposed that these alleles might directly impact DNA methylation at R2, consequently affecting *ADIPOQ* expression [204]. It is worth noting that in a previously conducted study, Bouchard et al. investigated whether DNA methylation of the placental *ADIPOQ* gene reflects the mother’s blood glucose level. They showed that the same promoter variant rs17300539 (c.–11391 G>A) is significantly associated with methylation of the C1 CpG island on the fetal side of the placenta [205]. Confirmation of the above studies was also found in the study by Zhang et al. It shows that increased methylation of the *ADIPOQ* in the same (R2) promoter region is associated with elevated fasting plasma glucose in visceral adipose tissue from obese individuals, including those with T2DM. They also demonstrated a positive correlation between BMI and *ADIPOQ* promoter methylation [211]. Houshmand-Oeregaard et al. assessed DNA methylation in a distinct proximal promoter region of *ADIPOQ* within subcutaneous adipose tissue. The analyzed group was adult offspring of women with gestational diabetes (GDM) versus controls (offspring of normoglycemic pregnancies). They targeted two CpG sites (–112 bp and –45 bp upstream of the transcription start site) and found significantly higher methylation in the O-GDM group. Furthermore, methylation at these positions positively correlated with BMI, HOMA-IR, fasting plasma glucose, and systolic blood pressure [212]. Another study conducted by Ott et al. examined DNA methylation across three regions within the *ADIPOQ* locus: a distal promoter region (R1), located approximately 5 kb upstream of exon 1; an intronic segment (R3), situated between exons 1 and 2 within the first intron; and an intergenic element (R2), which serves as a transcription factor binding site and is located within the gene body (the same R2 region was classified by Kim et al. and Zhang as a distal promoter) and revealed that the most pronounced hypermethylation occurred in the intronic R3 region, with an increase of up to 4% in visceral adipose tissue and peripheral blood cells. Notably, R2 and R3 methylation levels demonstrated a strong inverse correlation with *ADIPOQ* transcript abundance in adipocytes. This confirms the functional impact of these modifications on gene expression [213]. Houde et al. provided additional evidence that methylation of the proximal region of the *ADIPOQ* gene (CpGE1, located approximately –467 to –435 bp upstream of the transcription start site) in human adipose tissue correlates with features of metabolic syndrome, especially obesity severity and dyslipidemia. In their cohort of severely obese patients, CpGE1 methylation in subcutaneous adipose tissue positively correlated with BMI and waist circumference. In addition, the methylation in visceral fat tissue is positively correlated with elevated LDL cholesterol. These findings support the idea that *ADIPOQ* hypermethylation is both a biomarker and an active contributor to obesity-related metabolic dysfunction [214]. What is worth noting, Yi et al. demonstrated that excess nutrients, insulin, and insulin-like growth factor 1 (IGF-1) activate S6K1, serine/threonine kinase. Once activated, S6K1 translocates to the nucleus and phosphorylates histone H2B at serine 36. This modification recruits the polycomb repressive complex 2 (PRC2) subunit EZH2, which trimethylates histone H3 at lysine 27 (H3K27me3) in the promoter region of the *ADIPOQ* gene. This histone modification causes chromatin condensation in this regulatory region, which silences adiponectin transcription and reduces mRNA and protein levels. Alternatively, inhibiting S6K1—through rapamycin treatment or fasting—removes these repressive marks, which restores adiponectin expression and improves insulin sensitivity and glucose metabolism [206].

### 4.2. MicroRNA Regulation of ADIPOQ

Beyond DNA methylation, it has been shown that several miRNAs can modulate adiponectin levels by binding directly to the 3′ UTR of *ADIPOQ* mRNA or through indirect pathways. Ishida et al. demonstrated that miR-378 binds to a specific site in the mouse Adipoq 3′ UTR, decreasing Adipoq mRNA and adiponectin protein levels in adipocytes. A luciferase reporter assay confirmed that disrupting the binding site of miR-378 eliminated this repression. Furthermore, the inflammatory cytokine TNFα increases miR-378 levels, correlating inversely with Adipoq transcripts and protein levels. In leptin-deficient (ob/ob) mice, miR-378 levels are significantly higher in white adipose tissue than in wild-type controls, and they inversely correlate with Adipoq mRNA abundance. These data identify miR-378 as a critical link between inflammation and metabolic dysfunction [207]. Similarly, miR-221 targets the 3′ UTR of human *ADIPOQ* mRNA in adipocytes, destabilizing the transcript and reducing adiponectin secretion. In obese and T2DM diabetic adipose tissue, miR-221 levels correlate with higher BMI, increased HOMA-IR, and lower circulating adiponectin levels. In cultured adipocytes, inhibiting miR-221 restores *ADIPOQ* expression and enhances insulin-stimulated glucose uptake [215,216]. Conversely, Belarbi et al. discovered that overexpressing miR-193b in human subcutaneous adipocytes increases *ADIPOQ* mRNA and adiponectin release. MiR-193b does not directly bind the *ADIPOQ* 3′-UTR; instead, it targets the 3′-UTRs of the transcriptional repressors NF-YA and NRIP1. This reduces their levels, thereby relieving the repression of the *ADIPOQ* promoter. In obesity, miR-193b is downregulated, and its reduced expression correlates with lower *ADIPOQ* mRNA and protein [217]. Another indirect mechanism includes miRNA-34a, which enhances pro-inflammatory nuclear factor kappa B (NF-κB) signaling in adipose tissue. This leads to repressive histone modifications (decreased H3K27ac and increased H3K27me3) at the adiponectin locus. Elevated levels of miR-34a in obese individuals are associated with lower adiponectin levels, higher triglyceride levels, lower HDL-C levels, and impaired insulin resistance. In obese mice, treatment with anti-miR-34a increases adiponectin levels, improves insulin sensitivity, and reduces hepatic fat accumulation [218]. Additionally, miR-27a and its paralog, miR-27b, reduce PPARγ binding to the *ADIPOQ* promoter, thereby reducing transcription. In subcutaneous adipose tissue from patients with metabolic syndrome, levels of miR-27a correlated positively with BMI and inversely with serum adiponectin. In obese mouse models, inhibiting miR-27a increases PPARγ and adiponectin levels, improving lipid profiles and insulin sensitivity [219,220].

## 5. Modifiable and Non-Modifiable Determinants of Adiponectin Concentrations

Adiponectin plays a crucial role in the regulation of metabolic homeostasis [28]. Reduced circulating levels of this adipokine have been strongly associated with various components of metabolic syndrome, such as obesity, type 2 diabetes mellitus, hypertension, atherosclerosis, and coronary artery disease [221]. While genetic factors contribute to individual variations in its concentrations, a range of modifiable and non-modifiable factors, including age, sex, ethnicity, body mass index, dietary patterns, and physical activity levels, also significantly influence adiponectin expression and secretion [221,222].

Gender is a significant determinant of adiponectin levels [28]. Evidence consistently demonstrates that females tend to exhibit higher circulating concentrations of this adipokine than males, potentially attributable to disparities in sex hormone profiles [222,223]. Androgens, which are more abundant in males, have been shown to suppress adiponectin expression [221], whereas estrogens appear to exert a stimulatory influence on adipose tissue function [28]. Furthermore, elevated testosterone levels have been associated with the inhibition of HMW adiponectin production [27]. Ostrowska et al. observed markedly higher adiponectin concentrations in women (41.3 ± 20.1 μg/mL) compared to men (22.0 ± 14.8 μg/mL in men; *p* < 0.05) [221]. Similarly, findings by Cnop et al. confirmed a sex-related disparity, reporting levels of 7.4 ± 2.9 μg/mL in females and 5.4 ± 2.3 μg/mL in males (*p* < 0.0001) [224].

Dietary patterns, specific nutrients, and total caloric intake are critical factors influencing serum adiponectin concentrations. Balanced diets abundant in bioactive compounds—such as omega-3 fatty acids, dietary fiber, and polyphenols—have been shown to enhance adiponectin secretion and exert anti-inflammatory effects [222,225]. Dietary interventions including the Mediterranean diet, the DASH (Dietary Approaches to Stop Hypertension) diet, and plant-based diets are particularly effective, as they emphasize high consumption of vegetables, fruits, whole grains, legumes, olive oil, and fatty fish [222]. These eating patterns also typically encourage moderate alcohol intake and limit the consumption of red and processed meats, thereby contributing to their overall metabolic benefits [222]. The favorable impact of such dietary approaches on adiponectin levels has been well documented [49,226,227,228].

Low-calorie diets have been shown to effectively increase adiponectin levels. A meta-analysis revealed that adherence to such dietary regimens for up to 16 weeks results in a significant elevation of serum adiponectin concentrations (*p* < 0.01). This effect is partially attributed to the activation of the PPARγ signaling pathway, which enhances adiponectin gene expression. Moreover, the positive impact of low-calorie diets on adiponectin levels is further potentiated when combined with regular physical activity [229]. Additionally, low-carbohydrate diets, defined as those providing less than 30% of total energy from carbohydrates, have been investigated for their role in adiponectin modulation. Findings indicate that these diets induce a modest yet statistically significant increase in adiponectin concentrations (0.12 mg/mL, 95% CI 0.07–0.18) [230].

Omega-3 fatty acids, particularly those obtained from fish or dietary supplements, have been demonstrated to enhance adiponectin levels [28,127,222,225]. This effect is partly mediated through PPARγ activation, enhancing gene expression related to adiponectin production. Additionally, omega-3 fatty acids exert anti-inflammatory effects by downregulating pro-inflammatory cytokines such as TNF-α and IL-6 [222]. Silva et al. reported that regular consumption of fish or omega-3 supplements resulted in a 14–60% increase in circulating adiponectin concentrations [225]. These findings are further supported by a meta-analysis of 14 randomized controlled trials involving individuals with prediabetes and diabetes, which demonstrated a significant increase in adiponectin levels following omega-3 supplementation (0.48 μg/mL; 95% CI 0.27 to 0.68; *p* < 0.00001) [231]. Similarly, dietary fiber—particularly from whole grains and legumes—has been associated with elevated adiponectin concentrations [225]. Moreover, polyphenols found in coffee, green tea, berries, and curcumin have been found to stimulate adiponectin production, likely through PPARγ activation and attenuation of inflammatory pathways [222].

In contrast, Western dietary patterns, characterized by high consumption of saturated and trans fats, refined carbohydrates, and processed foods, have been consistently linked to decreased adiponectin concentrations [222]. These observations support the hypothesis that the westernization of dietary habits and lifestyle may negatively influence both the quantity and functional quality of adiponectin, thereby contributing to the development of insulin resistance. For example, comparative studies between Japanese-Americans and native Japanese individuals have demonstrated significantly lower adiponectin levels among those adhering to a Westernized diet [232].

Regular physical activity is a well-established regulator of serum adiponectin concentrations. Evidence suggests that consistent exercise not only promotes adiponectin secretion but also enhances its bioactivity, thereby contributing to improved metabolic outcomes. Interventions involving weight loss, physical activity, and dietary modification have been associated with elevated adiponectin levels [127]. For instance, one study reported a 51% increase in serum adiponectin following a 16-week moderate-intensity exercise program performed two to three times per week, although no significant changes were noted at the 8-week midpoint [127]. These findings are consistent with broader research indicating that physical activity elevates circulating adiponectin levels and enhances insulin sensitivity.

Although traditionally classified as an adipokine predominantly secreted by adipose tissue, emerging evidence indicates that adiponectin is also expressed in skeletal muscle, where it may exert autocrine and paracrine effects. Krause et al. reported a positive correlation between physical activity and both the adiponectin gene expression and circulating levels [223]. In contrast, sedentary behavior, obesity, and impaired glucose metabolism, characteristic of prediabetic and diabetic conditions, are typically linked to reduced adiponectin concentrations. Notably, structured exercise interventions have been shown to restore plasma adiponectin levels while simultaneously enhancing metabolic parameters such as insulin sensitivity [223]. These findings are further supported by a meta-analysis conducted by Yu et al., which assessed randomized controlled trials involving overweight and obese individuals. The analysis demonstrated that exercise significantly increased serum adiponectin concentrations by 0.44 μg/mL and concurrently reduced leptin levels by 2.24 ng/mL compared to control groups [233]. These alterations are clinically meaningful, as low adiponectin and elevated leptin levels have been linked to an increased risk of chronic diseases such as T2DM, CVD, and certain cancers.

## 6. Conclusions

This review aimed to present the role of adiponectin and genetic variants of the *ADIPOQ* gene in the development of metabolic syndrome and its components. Numerous studies have shown that reduced adiponectin levels are associated with obesity, insulin resistance, dyslipidemia, hypertension, and an increased risk of type 2 diabetes and cardiovascular disease. Particular attention was given to specific *ADIPOQ* variants that influence adiponectin multimerization, secretion, and biological activity, thereby contributing to metabolic disturbances. Understanding the genetic regulation of adiponectin offers valuable insight into metabolic homeostasis and may support the development of new therapeutic strategies for metabolic disorders. However, further research is needed to clarify the clinical relevance of individual variants and their interaction with environmental and lifestyle factors. Common promoter and intronic variants in the ADIPOQ gene change baseline adiponectin and modify how well patients respond to diet or insulin-sensitizing drugs; at the same time, first-generation adiponectin-receptor agonists have reached early-phase trials. Together, these advances outline a pragmatic route to genotype-guided therapy that could match “low-adiponectin” genotypes with receptor-targeted drugs now entering the clinic. However, further research is needed to clarify the clinical relevance of individual variants and their interaction with environmental and lifestyle factors.

## Figures and Tables

**Table 1:** Impact of *ADIPOQ* gene SNVs on obesity.

rsID	Allele Change	Variant Location/Molecular Consequences	Observed Associations	Study Population	References
rs182052	G > A	Intron	- A allele associated with obesity risk [OR = 1.56 (95% CI 1.12–2.19), *p* = 0.009], higher body weight (*p* = 0.009), BMI (*p* = 0.022), and WC (*p* = 0.005)	- Brazilian adults (n = 332)	[81]
			- Associated with WC (*p* = 0.0029) and serum adiponectin level in whites (*p* < 0.005); subjects with AA genotype had 15.6% lower (95% CI −21.3%, −9.4%) serum adiponectin per SD increase in WC versus AG genotype ↓22.8% (95% CI −25.4%, −20.1%) and GG genotype ↓16.9% (95% CI −19.7, −14.0)	- African-American and white adults from the US (n = 3355)	[82]
			- Associated with BMI (*p* = 0.037), WC (*p* = 0.009), WHR (*p* = 0.029), and SAT (*p* = 0.051)	- Hispanic adults (n = 811)	[83]
rs822393	C > T	Intron	- Associated with obesity [OR = 1.38 (95% CI 1.1–9.25), *p* = 0.037) and increased BMI (*p* = 0.028), WC, and HC	- Tunisian adults (n = 1121)	[84]
rs16861210	G > A	Intron	- No association with body weight; G allele associated with lower serum adiponectin concentrations (*p* = 0.029 for the additive and *p* = 0.008 for the dominant model)	- Finnish adults (n = 507)	[85]
			- No association with serum adiponectin level and BMI	- Black women (n = 990) and White women (n = 977) from the southeastern USA	[86]
rs822394	A > C	Intron	- No association with plasma adiponectin levels and adiposity phenotypes	- Hispanic (n = 1424) and African American (n = 604) population from the USA	[87]
			- No association with serum adiponectin levels and adiposity	- African-American adults from the USA (n = 2968)	[88]
rs822395	C > A	Intron	- Associated with obesity, homozygotes for the minor C allele were 50% less likely to be obese (OR = 0.51, 95% CI = 0.25–1.03). However, this association was no longer statistically significant after adjustment for rs1501299 (*p* = 0.21)	- African-American men from the USA: prostate cancer cases (n = 131) and controls (n = 344)	[89]
rs822396	G > A	Intron	- Associated with obesity risk as the GG genotype was significantly more frequent in the obese group (12.4%) than in the non-obese group (6%) (*p* < 0.008), and MetS parameters—GG genotype had higher mean values of BMI, TC, HDL-C, LDL-C, WC, WHR when compared to other genotypes	- North-Indian Punjabi adults (n = 550)	[90]
			- Associated with obesity risk (BMI, WC, visceral fat; *p* < 0.001) and MetS parameters (glucose, TG, TC, LDL-C; *p* < 0.001 and HDL-C; *p* = 0.008)	- Mexican adults (n = 434)	[91]
			- Association with generalized [OR = 1.38 (95% CI 1.02–1.87, *p* = 0.03) for GG genotype] and central obesity (significantly increased WC in GG genotype, *p* = 0.001); minor allele significantly associated with hypoadiponectinemia in normal glucose tolerance (6.3 ± 4.0 μg/mL, *p* < 0.0001), non-obese (7.5 ± 4.8 μg/mL, *p*< 0.0001), and obese patients (6.9 ± 4.4 μg/mL, *p* < 0.0001)	- South-Indian adults with normal glucose tolerance (n = 1100) and type 2 diabetes (n = 1100)	[92]
			- No association with adiponectin level or BMI	- Black women (n = 990) and White women (n = 977) from the southeastern USA	[86]
rs12495941	G > T	Intron	- Associated with obesity risk (OR = 2.373, SE = 0.2661, *p* = 0.001) but modulated by PEA	- African-American adults (n = 2968) from the USA	[88]
			- Associated with WC (*p* = 0.034) and glucose homeostasis (*p* = 0.022) only in Hispanic Americans	- Hispanic (n = 1424) and African American (n = 604) population from the USA	[87]
			- No association with adiponectin or BMI	- Black women (n = 990) and White women (n = 977) from the southeastern USA	[86]
rs2036373	T > G	Intron	- Associated with BMI (*p* = 0.032) and VAT (*p* = 0.022) in Hispanic Americans, not in African Americans; no association with adiponectin level	- Hispanic (n = 1424) and African American (n = 604) population from the USA	[87]
rs17366568	G > A	Intron	- No association with BMI; A allele associated with lower adiponectin level	- Finnish adults (n = 507)	[85]
			- No association with BMI; associated with higher serum adiponectin (adjusted mean adiponectin levels for GG = 15.9 mg/mL, AG = 13.7 mg/mL, AA = 9.3 mg/mL; *p* = 0.00036) in White women	- Black women (n = 990) and White women (n = 977) from the southeastern USA	[86]
			- No association with BMI; associated with higher SAT (*p* = 0.012) and WC (*p* < 0.001) in Hispanic Americans	- Hispanic (n = 1424) and African American (n = 604) population from the USA	[87]
			- AG and AA genotypes enriched in the obese group; association lost after Bonferroni correction; no association with circulating adiponectin	- Malaysian Malays (n = 574)	[93]
			- No association with obesity and any clinical parameters	- Tunisian adults (n = 1121)	[84]
rs17846866	T > G	Intron	- Significantly associated with a higher risk of obesity; TG genotype: OR = 1.53 (95% CI 1.30–1.80, *p* < 0.0001), GG genotype: OR = 2.10 (95% CI 1.30–3.30, *p* = 0.002)	- Asian Indian with T2D (n = 2000) and normal glucose tolerance (n = 2000)	[94]
			- Associated with increased BMI (*p* < 0.0001)	- Two Indian populations: Gujarat (n = 475 diabetic patients and n = 493 control subjects), Jammu and Kashmir (n = 507 diabetic patients and n= 300 controls)	[95]
rs2241766	T > G	Exon/synonymous variant	- Significantly associated with obesity: GG genotype OR = 3.52 (95% CI 1.81–6.85)	- Iranian adults: obese (n = 230) and healthy controls (n = 169)	[96]
			- Significantly associated with obesity in women: carriers of the TG or GG genotypes were 1.48 times more likely to be obese than TT carriers (95% CI 1.03–2.15, *p* = 0.036)	- Brazilian women from the PREDI Study (n = 435)	[97]
			- Associated with higher risk of overweight/obesity (OR = 3.07, 95%CI = 1.11–8.5; *p* = 0.03 for dominant model)	- Mexican women with primary breast cancer (n = 177)	[98]
			- No association with obesity in the studied cohort	- Turkish Cypriots: obese (n = 100) and non-obese (n = 100)	[99]
			- GG and GT genotypes associated with higher BMI compared to TT (*p* = 0.02)	- German nondiabetic individuals (n = 371)	[100]
			- GG genotype associated with obesity in Chinese studies (OR = 1.54, 95% CI 1.19–2.00) not in non-Chinese studies in adults	- Meta-analysis of 18 case-control studies	[101]
			- G allele was negatively associated with risk of metabolically unhealthy obesity (OR = 0.55; 95% CI = 0.40–0.75; *p* < 0.001 for additive model)	- Chinese adults: obesity phenotypes (n = 589) and healthy controls (n = 336)	[102]
			- No association with obesity in the studied population	- Kazakh adults with T2D (n = 136) and healthy adults (n = 577)	[103]
			- No association with obesity in the studied population	- Tunisian adults (n = 1121)	[84]
			- G allele was associated with obesity (OR = 2.50, 95%CI: 3.1–5.40; *p* < 0.0001)	- Pakistani subjects (n = 4000)	[104]
			- GT genotype was associated with the highest BMI (OR = 3.81, 95%CI: 1.79–8.09)	- Adults from Simferopol: patients with metabolic syndrome (n = 207) and healthy controls (n = 100)	[105]
			- No association with obesity risk	- Meta-analysis of 12 studies	[106]
rs1501299	G > T	Intron	- G allele was significantly associated with higher body weight at baseline (*p* = 0.032) and at 4-year follow-up (*p* = 0.041)	- Finnish adults with impaired glucose tolerance, all overweight (n = 507)	[85]
			- Significantly associated with higher BMI; additive model *p* = 0.03, TT homozygotes had more than twice the risk of obesity relative to GG (TT vs. GG: OR = 2.29, 95% CI 1.12–4.72, *p* = 0.02)	- African-American men: prostate cancer cases (n = 131) and controls (n = 344)	[89]
			- No association with higher BMI, T2DM, or serum adiponectin levels	- Pima Indian adults (n = 1338) 59% with T2DM and mean BMI ≈36.6 kg/m²	[107]
rs2241767	A > G	Intron	- G allele was significantly associated with central obesity based on WC, adjusted OR for AG vs. AA = 1.42, 95% CI = 1.12–1.81, *p* = 0.004 - Minor allele was significantly associated with the higher level of adiponectin in normal glucose tolerance (10.2 ± 6.5 μg/mL, *p* = 0.001), non-obese (9.5 ± 3.4 μg/mL, *p* = 0.02) and obese patients (9.4 ± 3.2 μg/mL, *p* < 0.0001) (*p* ≤ 0.001)	- South-Indian adults with normal glucose tolerance (n = 1100): obese (n = 818) and non-obese (n = 896)	[92]
			- No association with higher BMI, WC, or WHR; G allele was significantly associated only with the higher sum of skinfold thickness (*p* = 0.013)	- Mexican-American adults (n = 439) (116 with T2DM, 323 without T2DM)	[108]
rs3821799	T > C	Intron	- C allele was significantly associated with higher body weight (additive model, CC vs. CT vs. TT; *p* = 0.001, - F R q = 0.024)	- Finnish adults with IGT (n = 507)	[85]
			- No association with BMI and serum adiponectin levels	- British adult female twins (monozygotic and dizygotic), “Twins UK” cohort, n ≈ 2700	[109]
rs3774261	A > G	Intron	- Significantly associated with obesity only when combined with rs2241766 (*p* = 0.004). The association was significant after multiple-test corrections	- Belgian adult females with obesity, n = 223 and without obesity, n = 87	[110]
			- No association with higher BMI or with serum adiponectin levels	- African-American adults (n = 273)	[111]
			- No association with higher BMI; across all subgroups—with normal-glucose tolerance, T2DM, non-obese, and obese—A-allele carriers had significantly higher adiponectin concentrations in normal glucose tolerance (12.1 ± 5.3 μg/mL, *p* < 0.0001), non-obese (11.0 ± 5.2 μg/mL *p* < 0.0001) and obese patients (10.2 ± 4.5 μg/mL, *p* < 0.0001)	- South Indian adults with type 2 diabetes (n = 1100) and normal glucose tolerance (n = 1100), stratified into non-obese (BMI < 25 kg/m²) vs. obese (BMI ≥ 25 kg/m²) and low- vs. high-risk waist circumference groups	[92]
			- No association with higher BMI	- Malaysian adults with obesity (n = 150) and without obesity (n = 424)	[93]
rs3774262	G > A	Intron	- An allele was significantly associated with lower weight, WC, HC, and BMI compared with the GG genotype (ANOVA *p* = 0.012; trend *p* = 0.004)	- Chinese women (n = 1028) with newly diagnosed endometrial cancer and controls (n = 1932)	[112]
rs62625753	G > A	Intron	- No association with higher BMI; however, following a 4-week hypocaloric diet–exercise intervention, GA heterozygotes achieved larger reductions in TC (*p* = 0.010) and LDL-C (*p* = 0.036) and a greater rise in adiponectin (*p* = 0.005) compared with wild-type homozygotes	- Caucasian adults with severe obesity (n = 268; 87 males, 181 females) and healthy controls (n = 150; 49 males, 101 females)	[113]
rs17366743	T > C	Exon/missense variant	- No association with higher BMI was detected	- Finnish adults with IGT, all overweight (n = 507)	[85]
			- No association with obesity risk	- Italian Caucasian adults with severe obesity (n = 268; 87 males, 181 females) and healthy controls (n = 150; 49 males, 101 females)	[113]
			- No association with obesity risk, obesity, or adiponectin levels	- Framingham offspring study: predominantly European-ancestry men and women from the USA (n = 2543) followed for 28 years (1971–2002)	[114]
rs6444174	C > T	3′UTR	- No association with overall obesity risk; among normal-weight individuals (BMI < 30); the T allele was associated with lower BMI (*p* = 0.00036), whereas no association was observed in overweight/obese participants	- African-American adults from the Jackson Heart Study (n = 2968; 1131 men, 1837 women; ~83% of men and ~89% of women overweight/obese)	[88]
rs6773957	A > G	3′UTR	- G allele associated with higher BMI at baseline and after 4-year follow-up, and with greater body weight than the AA genotype (*p* = 0.033 or 0.009, model-dependent); G allele was also significantly associated with lower circulating adiponectin compared with AA (*p* = 0.016–0.022 across models)	- Finnish adults with IGT and overweight (n = 507)	[85]
			- G allele associated with a higher risk of a metabolically unhealthy phenotype (OR = 1.26, 95% CI 1.07–1.47, *p* = 0.004), with no association with BMI, WC, or other obesity indices, and linked to lower circulating adiponectin	- Chinese Han children/adolescents (n = 3317), analyzed separately as normal-weight and overweight/obese groups	[115]
rs2082940	T > C	3′UTR	- No association with higher BMI; T allele tended to associate with higher adiponectin, though it was not strongly significant (*p* = 0.056)	- Finnish adults with IGT and overweight (n = 507)	[85]
rs1063538	T > C	3′UTR	- No association with BMI or circulating adiponectin levels	- Pima Indian adults with overweight (n = 1338; 59% with T2DM)	[107]
rs1063539	G > C	3′UTR	- No association with BMI, bodyfat %, body weight, or WC	- Mexican adult volunteers from Mérida, Yucatán (n = 259)	[116]
			- No association with BMI was observed, and no significant association with circulating adiponectin levels was detected in either Black or White women	- African-American women (n = 990) and Caucasian women (n = 977) from the USA	[86]
			- G allele associated with higher weight, WC and BMI (GG > CG > CC; additive trend: weight *p* < 0.001, BMI *p* = 0.004); C allele associated with lower weight and BMI (weight: ANOVA *p* = 0.002, trend *p* < 0.001; BMI: ANOVA *p* = 0.012, trend *p* = 0.004)	- Chinese Han women (n = 1028) with endometrial-cancer and controls (n = 1932)	[112]
rs9842733	A > T	3′UTR	- No association with higher BMI or with serum adiponectin levels	- African-American women (n = 990) and Caucasian women (n = 977) from the USA	[86]
			- No association with higher BMI	- African-American men and women (n = 3020)	[117]
rs17300539	G > A	Promoter	- No association with obesity risk; A allele associated with higher circulating adiponectin (*p* = 0.005 in obese children, *p* = 0.00007 in general-population children)	- French Caucasian children: obese (n = 1229) and non-obese (n = 1350)	[118]
			- A allele associated with higher obesity risk in the pooled child population (additive model: OR = 1.33, 95% CI 1.03–1.71, *p* = 0.023); GA + AA carriers had a higher mean BMI by 0.97 kg m⁻² than GG after adjustment for age, sex and pubertal stage (*p* = 0.015); 1.6 µg/mL higher adiponectin levels in GA + AA carriers compared to GG carriers (*p* < 0.0001)	- European children with obesity (n = 519) and without obesity (n = 1333)	[119]
			- A allele associated with higher obesity risk (OR = 1.68, 95% CI 1.04–3.11, *p* = 0.044)	- Tunisian Arab adults with obesity (n = 160) and without obesity (n = 169)	[120]
rs266729	C > G	Promoter	- G allele was significantly associated with higher mean weight (*p* = 0.025); effect stronger in women than in men	- Finnish adults with IGT and overweight (n = 507)	[85]
			- G allele elevates risk of composite obesity (high BMI + low adiponectin; OR = 1.48, 95% CI 1.13–1.94) but shows no association with the BMI or adiponectin alone	- Koreans adults (n = 986) with overweight (n = 259) and without overweight (n = 272)	[121]
			- C allele was significantly associated with the obese risk OR = 1.23, 95% CI = 1.08–1.39, *p* = 0.001	- French Caucasians children with severe obesity (n = 1229) and without obesity (n = 1350)	[118]

**Abbreviations: ADIPOQ**—adiponectin gene; **SNV**—single-nucleotide variant; **BMI**—body mass index; **WC**—waist circumference; **WHR**—waist-to-hip ratio; **SAT**—subcutaneous adipose tissue; **HC**—hip circumference; **MetS**—metabolic syndrome; **PEA**—proportion of European ancestry; **VAT**—visceral adipose tissue; **TG**—triglycerides; **OR**—odds ratio; **CI**—confidence interval; **T2DM**—type 2 diabetes mellitus; **TC**—total cholesterol; **LDL-C**—low-density lipoprotein cholesterol; **IGT**—impaired glucose tolerance; **UTR (3′)**—3′ untranslated region.

**Table 3:** Impact of *ADIPOQ* gene SNVs on hypertension.

rsID	Allele Change	Variant Location/Molecular Consequences	Observed Associations	Study Population	References
rs12495941	G > T	Intron	- Noassociation with blood pressure	Chinese adults (n = 334)	[196]
			- No association with blood pressure	Chinese adults (n = 1616)	[197]
rs182052	G > A	Intron	- No association with blood pressure	Chinese adults (n = 334)	[196]
			- No association with blood pressure	Chinese adults (n = 1 616)	[197]
rs2241766	T > G	Exon/synonymous variant	- No association with hypertension	- Chinese Han adults: T2DM (n = 188), hypertension (n = 223), both T2DM + hypertension (n = 181), and controls (n = 176); total n = 768	[150]
			- Associated with resistant hypertension in young-onset hypertensive subjects	- Taiwanese adults with young-onset hypertension (n = 861)	[198]
			- No association with hypertension overall; G was allele protective in the high-TC subgroup (OR = 0.46, 95% CI 0.26–0.82, *p* = 0.009)	- Chinese adults from North China: cases (n = 296) and controls (n = 296)	[199]
rs1501299	G > T	Intron	- Marginally significant association with hypertension under a heterogeneous co-dominant model (TG vs. GG; OR = 0.86, 95% CI 0.75–0.99) and dominant model (TT + TG vs. GG; OR = 0.85, 95% CI 0.74–0.98)	- Meta-analysis of 4 studies	[195]
			- No association with hypertension risk	- Meta-analysis of 10 studies	[194]
			- SNV associated with decreased risk of hypertension in the allelic, dominant, and heterozygote models only in the Caucasian subgroup	- Meta-analysis of 9 studies	[193]
			- Allele T marginally related to hypertension risk	- Chinese adults from North China: cases (n = 296) and controls (n = 296)	[199]
			- SNV associated with the presence of hypertension only in the MetS group (OR for GG + GT vs. TT = 2.46; 95% CI 1.14–5.3, *p* = 0.02)	- 962 subjects from the Taiwan young-onset hypertension genetic study	[200]
rs822394	A > C	Intron	- Allele A was significantly associated with basal DBP in the recessive model (AA vs. AC + CC) with *p* = 0.033	- Chinese adults (n = 334)	[196]
rs266729	C > G	Intergenic	- G allele marginally associated with hypertension risk	- Chinese adults from North China: cases (n = 296) and controls (n = 296)	[199]
			- G allele significantly associated with higher hypertension risk (OR = 1.49, 95% CI 1.13–1.95)	- Chinese adults (n = 1 616)	[197]
			- G allele significantly associated with higher hypertension risk (OR = 1.41, 95% CI 1.02–1.95, *p* = 0.042; additive model *p* = 0.043)	- Chinese Han adults: T2DM (n = 88), hypertension (n = 223), both T2DM + hypertension (n = 181) and controls (n = 176); total n = 768	[150]
			- Affect susceptibility to essential hypertension in individuals exposed to high levels of arsenic	- Chinese adults exposed to high arsenic levels (n = 699)	[201]
rs1656930	A > G	Intergenic	- Minor allele associated with higher systolic blood pressure and greater hypertension prevalence	- Japanese adults (n = 3975)	[202]

**Abbreviations: *ADIPOQ***—adiponectin gene; **CI**—confidence interval; **DBP**—diastolic blood pressure; **OR**—odds ratio; **SNV**—single-nucleotide variant; **T2DM**—type 2 diabetes mellitus.

## Abbreviations

The following abbreviations are used in this manuscript:

MetS	Metabolic syndrome
T2DM	Type 2 diabetes mellitus
CVD	Cardiovascular disease
SNV	Single-nucleotide variant
TGs	Triglycerides
LDL-C	Low-density lipoprotein cholesterol
ASCVD	Atherosclerotic cardiovascular disease
NAFLD	Non-alcoholic fatty liver disease
IDF	International Diabetes Federation
AHA	American Heart Association
NHLBI	National Heart, Lung, and Blood Institute
WHF	World Heart Federation
IAS	International Atherosclerosis Society
IASO	International Association for the Study of Obesity
WC	Waist circumference
HDL-C	High-density lipoprotein cholesterol
GWAS	Genome-wide association studies
cDNA	Complementary DNA
C1q	Complement component 1q
Acrp30	Adipocyte complement-related protein of 30 kDa
AdipoQ	Adipocyte-derived Protein Q
ApM1	Adipose most abundant gene transcript
GBP28	Gelatin-binding protein of 28 kDa
TNF-α	Tumor necrosis factor-alpha
HMW	High molecular weight
NASH	Non-alcoholic steatohepatitis
ALT	Alanine transaminase
AST	Aspartate aminotransferase
iPSCs	Induced pluripotent stem cells
NAS	NAFLD Activity Scores
IR	Insulin resistance
TC	Total cholesterol
FFA	Free fatty acids
VAT	Visceral adipose tissue
SAT	Subcutaneous adipose tissue
BMI	Body mass index
GLP-1RA	Glucagon-like peptide receptor agonist
GPI-PLD	Glycosylphosphatidylinositol phospholipase D
CRP	C-reactive protein
SAA	Serum amyloid A
MHR	Monocyte to high-density lipoprotein cholesterol ratio
ECW/ICW	Extracellular to intracellular water ratio
AMPK	Adenosine monophosphate-activated protein kinase
PPAR-α	Proliferator-activated receptor-alpha
HFD	High-fat diet
RCTs	Randomized controlled trials
G6Pase	Glucose-6-phosphatase
PEPCK	Phosphoenolpyruvate carboxykinase
GLUT-4	Glucose transporter-4
S1P	Sphingosine 1 phosphate
IL-6	Interleukin-6
IL-8	Interleukin-8
NF-kB	Nuclear factor kappa-light-chain-enhancer of activated B cells
iNOS	Nitric oxide synthase
NADPH	Nicotinamide adenine dinucleotide phosphate
ACC	Acetyl-CoA carboxylase
CPT-1	Carnitine palmitoyltransferase 1
CD-36	Increasing cluster of differentiation 36
UCP-2	uncoupling protein-2
Acyl-CoA	Acyl-coenzyme A
ApoAI	Apolipoprotein AI
ABCA1	ATP-binding cassette transporter A1
LXRα	Liver X receptor alpha
PPARγ	Peroxisome proliferator-activated receptor gamma
HL	Hepatic lipase
VLDL	Very low-density lipoprotein
LPL	Lipoprotein lipase
ApoC-III	Apolipoprotein C-III
VLDLr	Very low-density lipoprotein receptor
IGT	Impaired glucose tolerance
SBP	Systolic blood pressure
DBP	Diastolic blood pressure
NO	Nitric oxide
eNOS	Endothelial nitric oxide synthase
VCAM	Vascular cell adhesion molecule-1
COX-2	Cyclooxygenase-2
RAAs	Renin-angiotensin-aldosterone system
IGF-1	Insulin-like growth factor 1
PRC2	Polycomb repressive complex 2
DASH	Dietary Approaches to Stop Hypertension
